# Language sentiment predicts changes in depressive symptoms

**DOI:** 10.1073/pnas.2321321121

**Published:** 2024-09-16

**Authors:** Jihyun K. Hur, Joseph Heffner, Gloria W. Feng, Jutta Joormann, Robb B. Rutledge

**Affiliations:** ^a^Department of Psychology, Yale University, New Haven, CT 06510; ^b^Department of Psychiatry, Yale University, New Haven, CT 06511; ^c^Wu Tsai Institute, Yale University, New Haven, CT 06510; ^d^Wellcome Centre for Human Neuroimaging, University College London, London WC1N 3AR, United Kingdom

**Keywords:** depression, symptom prediction, sentiment analysis, computational modeling

## Abstract

The prevalence of depression is a major societal health concern, and there is an ongoing need to develop tools that predict who will become depressed. Past research suggests that depression changes the language we use, but it is unclear whether language is predictive of worsening symptoms. Here, we test whether the sentiment of brief written linguistic responses predicts changes in depression. Across two studies (*N* = 467), participants provided responses to neutral open-ended questions, narrating aspects of their lives relevant to depression (e.g., mood, motivation, sleep). Participants also completed the Patient Health Questionnaire (PHQ-9) to assess depressive symptoms and a risky decision-making task with periodic measurements of momentary happiness to quantify mood dynamics. The sentiment of written responses was evaluated by human raters (*N* = 470), Large Language Models (LLMs; ChatGPT 3.5 and 4.0), and the Linguistic Inquiry and Word Count (LIWC) tool. We found that language sentiment evaluated by human raters and LLMs, but not LIWC, predicted changes in depressive symptoms at a three-week follow-up. Using computational modeling, we found that language sentiment was associated with current mood, but language sentiment predicted symptom changes even after controlling for current mood. In summary, we demonstrate a scalable tool that combines brief written responses with sentiment analysis by AI tools that matches human performance in the prediction of future psychiatric symptoms.

## Significance

Can the way we describe our life experiences predict whether we will feel more depressed in the future? We asked participants to write about their experiences from the past two weeks and complete a standard questionnaire assessing depressive symptoms, with a follow-up questionnaire administered three weeks later. Emotional tone of written responses assessed by human raters predicted changes in depression when controlling for both current depressive symptoms and current mood. AI tools matched human performance, suggesting an automated procedure for predicting future psychiatric symptoms from brief linguistic responses.

Depression is one of the major health and economic burdens in today’s society (1, 2). There is a growing emphasis in personalized medicine on efforts to predict whether depressive symptoms will worsen for specific individuals (3). One of the key challenges is to develop prediction tools that are both applicable in clinical settings and scalable to broad populations. As one promising approach, naturalistic language derived from social media or text messages has been proposed to track depression status (4). Individuals with depression used more negative emotional words on social media (5–8) and in text messages (9, 10). Language in a therapy context was also associated with treatment outcomes. Individuals using less self-referential language (i.e., fewer first-person singular pronouns) showed better treatment responses in a digital single-session intervention (11) and using a large telepsychotherapy platform (12).

Recent studies suggest that language use in depression depends on relevance to the self. Self-referential language derived from detailed clinical interviews predicts future depression scores for inpatients diagnosed with depression (13). Individuals with depression used more self-referential and negative emotional words when sending text messages to close contacts (14). Self-focused language was prominent when language was generated for prompts of a personal nature, momentary thoughts, or personal identity, but not for impersonal matters (15). Recent research also shows that Large Language Models (LLMs) trained on social media data predicted depressive symptoms assessed by the Patient Health Questionnaire (PHQ-9) (16), consistent with increasing potential for the use of LLMs for psychological research (17). Together, these studies suggest that it may be possible to build a tool that predicts changes in depression from language that does not require clinical interviews or access to social media or text messages. Such a tool combined with automated LLM-based analysis of self-referential text responses would be of considerable clinical and societal value.

We developed nine open-ended questions derived from the PHQ-9 (18), which itself is based on the diagnostic criteria for Major Depressive Disorder listed in the DSM-5 (19). We neutralized tone by removing negative framing words or phrases (e.g., depressed, feeling down, trouble concentrating), allowing written responses to reflect a range of emotional responses, whether positive, negative, or neutral. We collected depression-relevant narratives for on average less than 10 min. Given the already self-focused nature of the questions, we focused on analyses of the emotional tone or sentiment of responses. Specifically, we tested whether language sentiment related to depressive symptoms and predicted changes in symptoms at follow-up.

A popular approach to automatic sentiment analysis involves counting the number of emotional words in text using validated software like Linguistic Inquiry and Word Count (LIWC) (20–22). This approach operates on the assumption that each word is independent and sentiment ratings are not affected by word order (e.g., whether the positive words are at the beginning or end of a sentence). Recent studies have demonstrated that the LLM Generative Pre-trained Transformer (ChatGPT) developed by OpenAI (23) exhibits human-level performance in rating sentiment (24) and other benchmarks (25). Here, we test whether future depressive symptoms can be predicted by automatic sentiment ratings using LIWC-22 (version 1.8.0) and ChatGPT, comparing performance to the gold standard of human raters.

Given the connection between language sentiment and current mood (26), we also used computational modeling to rule out potential factors related to current mood dynamics that could explain any prediction accuracy. Computational modeling of data from decision-making tasks can capture latent cognitive and affective processes, each uniquely sensitive to individual psychiatric symptoms (27–30). Specifically, we focus on characterizing each individual’s affective states, such as current mood, and specific affective responses to good and bad news (i.e., affective reactivity). Here, we investigate whether language sentiment exhibits associations with mood dynamics and test whether any ability to predict future depressive symptoms is explained by mood-dependent model parameters from our task.

In this study, we find that transforming existing questionnaires into an open-ended format allows efficient language data collection and that linguistic features assessed by human raters or AI tools predict subsequent changes in psychiatric symptoms. We replicate previous findings that current mood assessed during a cognitive task and quantified using computational models predicts both depressive symptoms (31) and language sentiment (26). However, we find that only language sentiment and not current mood predicts changes in depressive symptoms. Overall, we demonstrate a scalable tool for efficiently collecting and analyzing symptom-relevant text responses that can be used in clinical settings.

## Results

### Language Sentiment Correlates with Future Depressive Symptoms.

We first examined whether language sentiment of, on average, less than 10 min of written responses to neutral open-ended questions relates to current and future depressive symptoms (Study 1, *N* = 179; Study 2, *N* = 288; *Materials and Methods* and *SI Appendix,* Fig. S1*A* and Table S1). We recruited human raters (*N* = 470) and asked them to rate a random subset of the collected written responses in terms of their affective tone (i.e., positivity and negativity; *Materials and Methods*) as the gold standard for sentiment ratings. Positive and negative sentiment ratings were highly correlated (Study 1, Spearman *ρ* = −0.90, *P* < 0.001; Study 2, *ρ* = −0.92, *P* < 0.001), and we used the difference between the positive and negative ratings from two human raters for each of the nine responses to create a composite sentiment score. Due to the non-normality of PHQ-9 scores in the general population (32, 33), we computed nonparametric Spearman correlations between sentiment ratings and PHQ-9 at initial and follow-up sessions (*Materials and Methods*). We also used robust linear regression to test whether sentiment predicted future depressive symptoms (i.e., PHQ-9 scores after three weeks), while accounting for initial depressive symptoms and important covariates (age, gender, and education). The sentiment expressed in written responses was negatively correlated with current depression measured by PHQ-9 (Study 1, *ρ* = −0.66, *P* < 0.001; Study 2, *ρ* = −0.58, *P* < 0.001), indicating that individuals with higher depression expressed more negative than positive sentiment in their text responses. Individual sentiment scores also correlated with future depressive symptoms measured three weeks later (Study 1, *ρ* = −0.61, *P* < 0.001; Study 2, *ρ* = −0.60, *P* < 0.001).

**Fig. 1.**
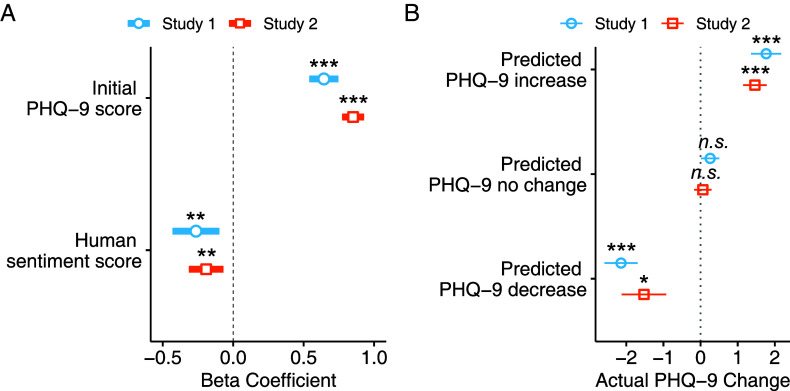
Sentiment ratings predict changes in depression. (*A*) The x-axis of the graph shows the beta coefficient from a robust linear regression model which included age, gender, and education level as covariates. The y-axis of the graph shows the predictors in the model (covariates not shown). In both studies (Study 1, blue line with a circle; Study 2, orange line with a square), more negative human-rated sentiment scores predicted higher depressive symptom scores (i.e., more depressed) at the three-week follow-up after controlling for initial depression (PHQ-9) scores. (*B*) For both studies, participants were grouped by whether no change in PHQ-9 was predicted by the model (predicted change between −0.5 and 0.5) or whether increased or decreased symptoms were predicted (Study 1, blue line with a circle; Study 2, orange line with a square). Error bars indicate the SEM. *P*-values indicate a significant difference from zero change using Wilcoxon signed-rank tests. *P < 0.05; **P < 0.01; ***P < 0.001.

However, since depression scores measured at different times are strongly correlated (34, 35), these findings do not show that language sentiment predicts changes in depression. Given that the current depression level was highly indicative of future depression in both studies (Study 1, *ρ* = 0.78, *P* < 0.001; Study 2, *ρ* = 0.84, *P* < 0.001), language sentiment might correlate with future depressive symptoms simply because future and current symptoms are correlated. Critically, we found that human-rated language sentiment is related to changes in depression: When individual written responses were rated as more negative at baseline, depression scores at follow-up increased compared to initial depression scores (Study 1, $\beta_{\mathrm{Human}R\mathrm{aters}}$ = −0.26, *SE* = 0.09, *t* = −3.11, *P* = 0.002, $f_{\beta_{\mathrm{Huma}nRaters}}^{2}$ = 0.051; Study 2, $\beta_{\mathrm{Human}R\mathrm{aters}}$ = −0.19, *SE* = 0.06, *t* = −3.07, *P* = 0.002, $f_{\beta_{\mathrm{Human}R\mathrm{aters}}}^{2}$ = 0.018; see Fig. 1*A*). For individuals whose depression scores were predicted to decrease, stay the same, or increase, the mean observed changes in depressive symptoms were on average significantly lower than zero, not different from zero, or greater than zero, respectively (see Fig. 1*B*). When modeled separately, human-rated positive and negative sentiment were both predictive of future depression (*SI Appendix,* Table S2).

To evaluate model performance, we conducted leave-one-out cross-validation while using the same robust regression model to predict changes in depression (Study 1, RMSE: 3.05, MAE: 2.17; Study 2, RMSE: 3.23, MAE: 2.26). We found significant correlations between predicted and actual changes in depression scores (Study 1, Pearson *R* = 0.43, *P* < 0.001; Study 2, *R* = 0.17, *P* = 0.004). In both studies, an ANOVA using the Wald test indicated a significant difference between the full model and the nested model without sentiment scores (Study 1, *W*(1) = 9.65, *P* = 0.002; Study 2, *W*(1) = 9.41, *P* = 0.002), indicating that inclusion of sentiment scores improved model fit.

We also tested whether sentiment predicts follow-up depression in individuals with higher or lower levels of initial depression. We split participants for Study 1 and 2 into separate groups depending on whether initial PHQ-9 scores were below five. Human-rated sentiment predicted future depressive symptoms above and beyond initial depression scores (*SI Appendix*, Fig. S6) in individuals with minimal symptoms ($\beta_{\mathrm{Human}R\mathrm{aters}}$ = −0.15, *SE* = 0.05, *t* = −2.95, *P* = 0.003) or with mild-to-moderate symptoms ($\beta_{\mathrm{Human}R\mathrm{aters}}$ = −0.29, *SE* = 0.09, *t* = −3.23, *P* = 0.001). When we only use subsets of sentiment ratings from different rater demographic groups (females or males, and older or younger), we continue to find that sentiment predicts depression trajectory (*SI Appendix*).

While our primary focus was on assessing sentiment in written responses that were already narratives about the self, we additionally investigated the relationship between depression and linguistic distance, which captures psychological distance from a self-focused and present-focused view (11, 12, 36–38). As reduced linguistic distance is associated with depression (11, 12), we examined this measure as another potential predictor of changes in depression. Using an established approach (12), we computed a composite linguistic distance score by averaging LIWC-based social distance (i.e., other-focused language) and temporal distance (i.e., nonpresent focused language) scores. The social distance score represents the proportion of non-first-person singular pronouns relative to all pronouns used in a response. Temporal distance was calculated as the proportion of nonpresent tense words relative to all tense words (see *SI Appendix* for details). We found a negative relationship between linguistic distance and current depressive symptoms in both Study 1 (*ρ* = −0.16, *P* = 0.028) and Study 2 (*ρ* = −0.11, *P* = 0.040), replicating previous findings (11, 12). However, within the context of brief self-relevant narratives, linguistic distance did not predict future depressive symptoms when controlling for current depression (*SI Appendix*, Fig. S2*A*). Human-rated sentiment remained a significant predictor of future depressive symptoms even after accounting for linguistic distance (*SI Appendix*, Fig. S2*B*). In exploratory analyses, we also tested whether increased use of self-focused language (i.e., greater first-person singular pronouns) was associated with stronger predictions of depression trajectories from human-rated sentiment scores but did not find consistent evidence for any interaction (*SI Appendix*, Table S3).

Notably, we found in two independent studies that more negative language sentiment predicted increased depressive symptoms three weeks later. In Study 1, we collected both written responses and depressive symptoms on the same day. In Study 2, depressive symptoms were measured at the initial session, and written responses were collected the following day. Predictions remained consistent whether written responses were provided on the same day as depression scores or on different days. This suggests that language sentiment measures primarily reflect trait-level processes that precede changes in depressive symptoms, rather than capturing transient processes that vary from day to day. This study demonstrates that language sentiment in brief written responses provides unique information about the trajectory of depression.

### Large Language Model Sentiment Ratings Predict Changes in Depression.

While sentiment ratings provided by human raters serve as the gold standard for the emotional tone of written responses, it is time-consuming and costly to obtain manual sentiment ratings from human raters. Scalability of this prediction pipeline would be enhanced by the application of automatic sentiment rating tools. To investigate whether cost-effective and automatic sentiment rating methods could replicate our results, we assessed the sentiment of written responses using two different tools, ChatGPT (GPT-3.5 model) and LIWC-22 (version 1.8.0). We instructed ChatGPT to provide two numerical ratings indicating the positive and negative tone of the written responses on a 0 to 10 scale (i.e., “How negative and positive is this text on a scale of 0 to 10?”) and, as with human raters, we combined these ratings into a single sentiment rating for each response that was then averaged across the nine responses into a sentiment score. A similar sentiment rating prompt has been used in previous research to show consistency between human raters and ChatGPT (24). We also found that the sentiment scores generated by ChatGPT for each individual were highly correlated with the human sentiment scores (Study 1, *ρ* = 0.96, *P* < 0.001; Study 2, *ρ* = 0.96, *P* < 0.001).

**Fig. 2.**
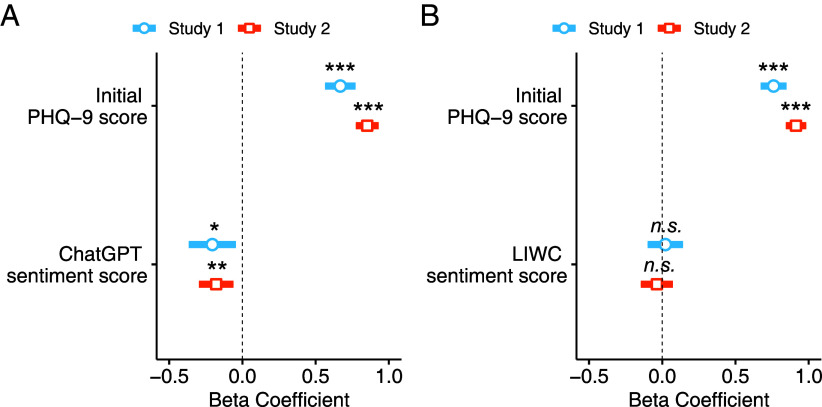
Sentiment ratings from LLMs, but not LIWC, predict future depression. (*A* and *B*) Robust linear regression predicting future depressive symptom scores (PHQ-9) after three weeks using automatic sentiment ratings by (*A*) ChatGPT and (*B*) LIWC. Note that in all models, the PHQ-9 scores at follow-up were used as a dependent variable, and age, gender, and education level were used as covariates. (*A*) In the analyses using ChatGPT sentiment scores, more negative sentiment scores predicted increased depressive symptoms (i.e., more depressed) at the three-week follow-up in both studies, after controlling for initial depression scores. (*B*) In analyses using LIWC sentiment scores, more negative sentiment scores did not predict increased depressive symptoms at the three-week follow-up in both studies, after controlling for initial depression scores. *P < 0.05; **P < 0.01; ***P < 0.001.

We found that human-rated language sentiment predicted changes in depression, and we tested whether this was also true for ChatGPT sentiment ratings. ChatGPT sentiment scores were negatively correlated with current depression in both Study 1 (*ρ* = −0.68, *P* < 0.001) and Study 2 (*ρ* = −0.59, *P* < 0.001). In robust linear regression analyses, ChatGPT sentiment scores consistently predicted future depressive symptoms after three weeks (Study 1, $\beta_{\mathrm{ChatGPT}}$ = −0.21, *SE* = 0.08, *t* = −2.50, *P* = 0.013, $f_{\beta_{\mathrm{ChatGPT}}}^{2}$ = 0.038; Study 2, $\beta_{\mathrm{ChatGPT}}$ = −0.18, *SE* = 0.06, *t* = −2.99, *P* = 0.003, $f_{\beta_{\mathrm{ChatGPT}}}^{2}$ = 0.018; see Fig. 2*A*), controlling for initial symptoms. Positive emotional tone of written responses rated by ChatGPT was associated with reduced PHQ-9 symptom scores (i.e., lower depression) after three weeks. In other words, individuals who wrote more negatively at the initial session experienced increased depressive symptoms at follow-up. We replicated these findings using ChatGPT model GPT-4 (*SI Appendix*, Figs. S3 and S4).

We compared our findings using ChatGPT sentiment measures with the most widely used automated emotional word count tool, LIWC. LIWC was used to calculate the proportion of positive and negative tone words in written responses as a measure of sentiment (20). LIWC sentiment scores were correlated with the human sentiment scores in both Study 1 (*ρ* = 0.75, *P* < 0.001) and Study 2 (*ρ* = 0.71, *P* < 0.001). Additionally, we observed similar correlations between LIWC and ChatGPT sentiment scores (Study 1, *ρ* = 0.76, *P* < 0.001; Study 2, *ρ* = 0.73, *P* < 0.001). We found a negative relationship between LIWC sentiment scores and current depression in both Study 1 (*ρ* = −0.52, *P* < 0.001) and Study 2 (*ρ* = −0.47, *P* < 0.001). However, LIWC sentiment scores did not predict future depression above and beyond initial depressive symptoms (Study 1, $\beta_{\mathrm{LIWC}}$ = 0.02, *SE* = 0.06, *t* = 0.34, *P* = 0.734, $f_{\beta_{\mathrm{LIWC}}}^{2}$ = −0.005; Study 2, $\beta_{\mathrm{LIWC}}$ = −0.04, *SE* = 0.06, *t* = −0.66, *P* = 0.511, $f_{\beta_{\mathrm{LIWC}}}^{2}$ = −0.002; Fig. 2*B*). As LIWC calculates the percentages of emotional tone words, insufficient total word counts can lead to less reliable sentiment scores. We addressed this psychometric concern by 1) applying data exclusion based on low word counts and 2) using modified LIWC scores computed from concatenated text responses (*SI Appendix*). In these subsequent analyses, LIWC sentiment did not predict changes in depression in either study (*SI Appendix*, Table S4 and Fig. S5).

While LIWC sentiment scores capture both emotionally salient (e.g., frustrated) and ambivalent-but-leaning words (e.g., noisy), language sentiment predicting future depression may be reflected mostly in emotionally salient words. To determine whether specific emotion words identified by LIWC could predict changes in depression, we conducted additional analyses using positive and negative emotion categories and emotion-specific categories, including sadness, anger, and anxiety (*SI Appendix,* Table S5). Although the LIWC negative emotion, anxiety, and anger categories predicted changes in depression in Study 1, these results did not replicate in Study 2 (*SI Appendix*, Table S5*A*). All findings remained consistent even after accounting for the LIWC sentiment scores (*SI Appendix*, Table S5*B*) or human-rated sentiment scores (*SI Appendix*, Table S5*C*). We additionally found that when LIWC positive and negative sentiment were modeled separately, they still did not predict changes in depression. In contrast, ChatGPT-rated positive and negative sentiment were individually predictive of future depressive symptoms beyond initial symptoms (*SI Appendix*, Table S2). Taken together, these results show that ChatGPT and human ratings consistently capture emotional information in text beyond the frequency of emotional tone words and that this information is predictive of changes in depressive symptoms.

### Language Sentiment Reflects Current Mood, but not Emotional Reactivity.

One of the underlying processes that language sentiment may reflect is ongoing mood dynamics. For example, individuals may narrate more negatively about past experiences when in a negative mood. Alternatively, individuals who are more reactive to negative emotional events may also write more negatively about their experiences. Either current mood or emotional reactivity could explain the predictive power of sentiment scores. To investigate this hypothesis, we collected happiness ratings during an established decision-making task (*Materials and Methods*) and applied an established computational model of momentary mood (39–41) to extract parameters for baseline mood and for emotional reactivity (i.e., emotional sensitivity to reward prediction errors).

**Fig. 3.**
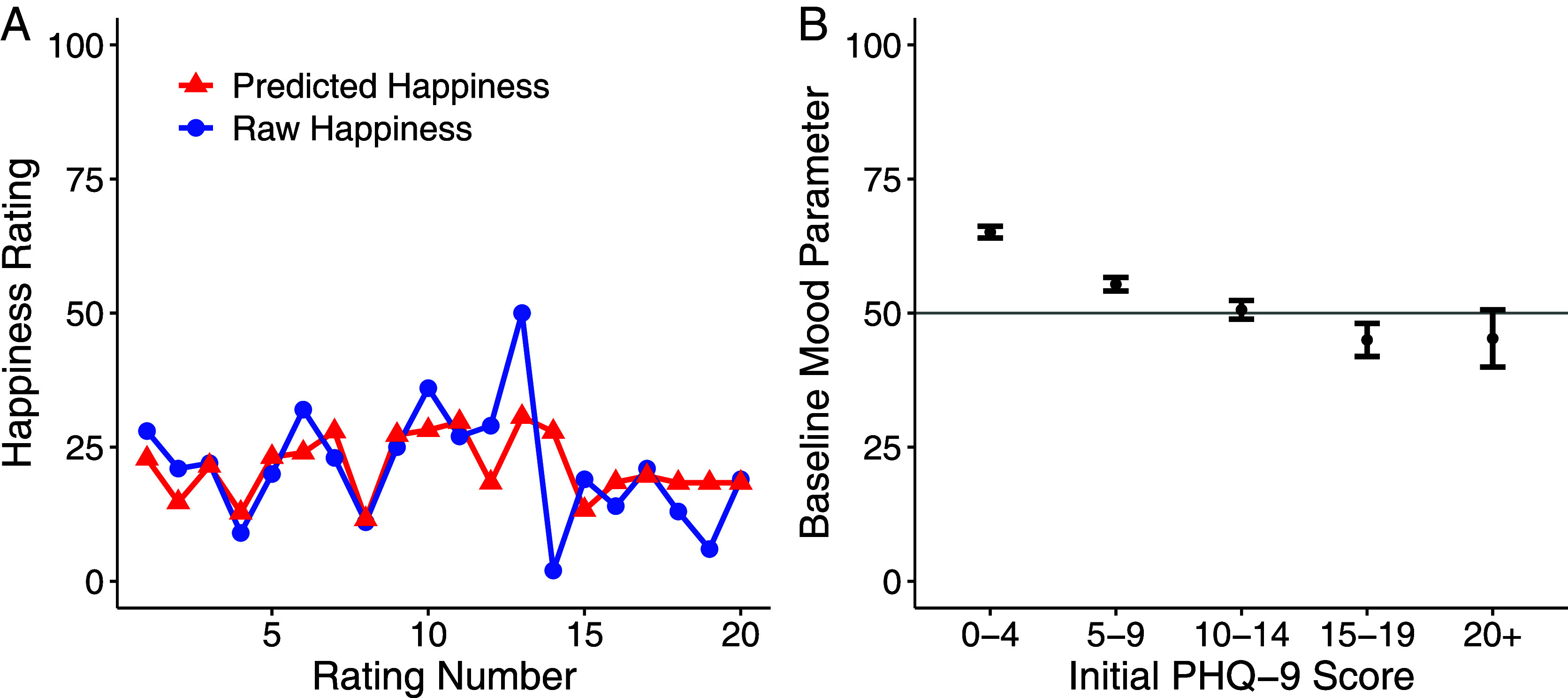
Momentary mood ratings are correlated with initial depressive symptoms. (*A*) In an example participant with the initial depression (PHQ-9) score of 22, a computational model of momentary mood accounted for subjective momentary mood ratings from the decision-making task. (*B*) Baseline mood parameters were negatively correlated with initial depressive symptoms (PHQ-9, *ρ* = −0.34, *P* < 0.001), replicating previous findings (31, 40). Error bars indicate the SEM. Study 1 and 2 datasets were combined for this analysis.

The computational model accounted for participant ratings in both studies (Study 1, mean $r^{2}$ = 0.53; Study 2, mean $r^{2}$ = 0.55; Fig. 3*A*). Using parameter estimates of baseline mood and emotional reactivity, we found that more positive (i.e., less negative) human-rated sentiment scores correlated with higher baseline mood parameters (Study 1, *ρ* = 0.40, *P* < 0.001; Study 2, *ρ* = 0.25, *P* < 0.001). This pattern remained consistent with both ChatGPT and LIWC sentiment scores (*SI Appendix,* Table S6). However, emotional reactivity did not correlate with human-rated sentiment scores in either study (Study 1, *ρ* = −0.08, *P* = 0.305; Study 2, *ρ* = −0.004, *P* = 0.946; *SI Appendix,* Table S6).

Baseline mood parameters were negatively associated with initial depressive symptoms (*ρ* = −0.34, *P* < 0.001; see Fig. 3*B*), consistent with previous findings (31). We used a robust linear regression model to investigate whether current mood remained associated with current depressive symptoms even when accounting for sentiment scores, age, gender, and education level. Our results were mixed, with baseline mood accounting for depressive symptoms after including sentiment scores in the model in Study 2 but not Study 1 (Study 1, $\beta_{\mathrm{Baseline}\mathrm{Mood}}$ = −0.01, *SE* = 0.01, *t* = −0.88, *P* = 0.379, $f_{\beta_{\mathrm{Baseline}\mathrm{Mood}}}^{2}$ = 0.001; Study 2, $\beta_{\mathrm{Baseline}\mathrm{Mood}}$ = −0.04, *SE* = 0.01, *t* = −2.90, *P* = 0.004, $f_{\beta_{\mathrm{Baseline}\mathrm{Mood}}}^{2}$ = 0.026).

### Language Sentiment Predicts Changes in Depression After Controlling for Mood.

Because current mood is highly related to language sentiment, it is possible that the baseline mood parameter also predicts changes in depressive symptoms. However, we found that the baseline mood parameter did not predict future depressive symptoms in a model without sentiment scores (Study 1, $\beta_{\mathrm{Baseline}\mathrm{Mood}}$ = −0.01, *SE* = 0.01, *t* = −0.94, *P* = 0.346, $f_{\beta_{\mathrm{Baseline}\mathrm{Mood}}}^{2}$ = 0.003; Study 2, $\beta_{\mathrm{Baseline}\mathrm{Mood}}$ = −0.01, *SE* = 0.01, *t* = −1.11, *P* = 0.267, $f_{\beta_{\mathrm{Baseline}\mathrm{Mood}}}^{2}$ = 0.0004). We then tested whether sentiment scores remained predictive of future depressive symptoms after accounting for baseline mood parameters. Human sentiment scores still predicted future depression in both studies, even when both baseline mood parameters and initial depression scores were accounted for (Study 1, $\beta_{\mathrm{Human}R\mathrm{aters}}$ = −0.26, *SE* = 0.09, *t* = −2.95, *P* = 0.004, $f_{\beta_{\mathrm{Human}R\mathrm{aters}}}^{2}$ = 0.045; Study 2, $\beta_{\mathrm{Human}R\mathrm{aters}}$ = −0.19, *SE* = 0.06, *t* = −2.91, *P* = 0.004, $f_{\beta_{\mathrm{Human}R\mathrm{aters}}}^{2}$ = 0.017). ChatGPT sentiment scores also predicted future depression beyond current mood and initial depression in both studies (Study 1, $\beta_{\mathrm{ChatGPT}}$ = −0.20, *SE* = 0.09, *t* = −2.33, *P* = 0.021, $f_{\beta_{\mathrm{ChatGPT}}}^{2}$ = 0.033; Study 2, $\beta_{\mathrm{ChatGPT}}$ = −0.17, *SE* = 0.06, *t* = −2.82, *P* = 0.005, $f_{\beta_{\mathrm{ChatGPT}}}^{2}$ = 0.017), whereas LIWC did not (Study 1, $\beta_{\mathrm{LIWC}}$ = 0.04, *SE* = 0.06, *t* = 0.64, *P* = 0.523, $f_{\beta_{\mathrm{LIWC}}}^{2}$ = −0.010; Study 2, $\beta_{\mathrm{LIWC}}$ = −0.03, *SE* = 0.06, *t* = −0.48, *P* = 0.629, $f_{\beta_{\mathrm{LIWC}}}^{2}$ = −0.002). Overall, language sentiment relates to current mood but is predictive of future depressive symptoms even after controlling for current mood.

## Discussion

To address the growing need for scalable tools to predict future changes in psychiatric symptoms, we introduce an approach that integrates clinically relevant open-ended responses with automated sentiment analysis tools. Remarkably, in two independent experiments, we find that open-ended responses that can be collected in less than 10 min contain critical information for forecasting depression three weeks in the future. More precisely, the emotional tone of the text, as assessed by both human evaluators and ChatGPT (at a total cost of less than $1 for analysis of the entire dataset), improves clinical predictions about future intensification or alleviation of depressive symptoms. While language sentiment is associated with current mood assessed with computational models, current mood does not predict changes in depression. In summary, our approach offers a significant advance in the precision of depression prognosis.

The main contribution of this study is that we not only replicate the known association between the sentiment of naturalistic language and depressive symptoms but extend this understanding to the prediction of changes in depression using brief open-ended questions that can be efficiently collected. Previous studies have used social media posts to show that linguistic features in social media precede depressive symptoms, the onset of depression, and the diagnosis of depression (5, 6, 42). Due to the nature of retrospective social media data collection, however, it has been challenging to gather initial levels of depression from the periods when the language data were provided. Prospective data collection has been applied to collect text messages with standardized symptom measures (9, 10), but the utility in clinic settings is unclear due to privacy concerns, invasiveness, and the duration of data collection. By collecting brief written responses that explicitly exclude identifiable information and standard depression questionnaires together, our study demonstrates that language sentiment correlates not just with future symptoms but more importantly with changes in symptoms. We find that a more negative sentiment expressed in writing predicts increases in depression scores after three weeks, even when accounting for the individual’s current depression level. Furthermore, we find that sentiment prediction of future symptoms is consistent when separately considering individuals with minimal or mild-to-moderate levels of initial depressive symptoms. Future research is needed to examine whether our approach can be used to monitor and predict symptoms in clinical populations diagnosed with depressive disorders including those with severe symptoms.

Automated language analysis tools offer richer ways to analyze linguistic data, which can be integrated with existing survey measures. Our results indicate that both LIWC and ChatGPT sentiment measures correlate with human-rated sentiment and that ChatGPT sentiment ratings are consistent with human ratings in predicting changes in depression. These findings underscore the potential of using automated text analysis tools to improve psychological assessments at minimal cost. Our analysis expenses were almost free, less than 0.1 cent per individual based on current GPT-3.5 prices. Although modeling the differences between dictionary-based and LLM-based sentiment analyses was not the primary focus of this study, understanding which linguistic features explain sentiment is an important question for future research. Customizable natural language processing techniques, such as topic modeling, can be used to identify cognitive factors (i.e., cognitive distortions), potentially revealing the aspects of sentiment that LLMs uniquely capture (43–45). Future research could also investigate other relevant semantic and syntactic factors, including the role of word order in sentiment ratings, and explore additional linguistic features linked to depression (46).

Our study strongly supports the broader adoption of open-ended self-description methods in psychological research. We found that open-ended questions, derived from multiple-choice questions, capture additional nuanced and individualized narratives associated with psychological symptoms like depression. This approach could be extended to a wide range of topics beyond the study of psychiatric disorders, including areas traditionally explored through self-report questionnaires, such as personality traits, social beliefs, and psychological well-being. Open-ended responses offer rich narratives that reveal how individuals understand, interpret, and respond to questions in self-report questionnaires, providing benefits over traditional Likert scale ratings. However, when adopting open-ended questions to gain insights into psychiatric symptoms, it is important to consider the potential challenges some individuals, particularly those with severe psychiatric symptoms, might encounter in providing written or spoken responses. For example, a small subgroup of individuals with poor clinical insight into their schizophrenia may underreport symptoms compared to clinician reports (47), leading to less accurate representations of their experiences. Our approach is meant to complement, not replace, existing methods, offering the potential to provide insights across a wide range of psychological and psychiatric questions.

To probe the relationship between language sentiment and mood dynamics, we included a decision-making task with happiness ratings. Sentiment could be implicated in both how we generally feel in a certain context and how we emotionally react to good and bad news. To dissect mood dynamics into these two components, we used an established computational model of happiness (31, 39–41). A more positive language sentiment is associated with higher current mood as captured by a higher baseline mood parameter but does not reflect either heightened or reduced emotional reactivity. Overall, current mood does not predict depression changes, but language sentiment predicts depression changes after controlling for current mood.

Despite these promising findings, our study has limitations. First, we did not test for long-term changes in depressive symptoms beyond three weeks. Previous research showed that the longer the time interval between social media posts and the date of depression diagnosis, the lower the predictive power of social media language (6). Second, the open-ended questions we developed asked about a limited range of individual experiences related to depression. The nine aspects of daily experiences covered by the questions were consistent with the nine diagnostic questions for Major Depressive Disorder in DSM-5. However, the list does not explore many other experiences linked to depression, such as social relationships. How the framing of these questions affects participants’ responses also remains an open question. By testing different framings of the open-ended questions and incorporating additional depression-relevant ones, we anticipate that prediction power can improve. While additional questions may require more time from participants, the burden associated with providing additional narrative responses is low, particularly if responses can be spoken. Automated dictionary- and LLM-based sentiment analyses are essentially free.

In summary, we propose that sentiment from brief written responses is predictive of changes in depressive symptoms and automated analysis tools can serve as an efficient way to quantify language sentiment. Overall, our findings highlight the potential for language sentiment analysis to provide valuable insights into the dynamics of depression and offer rich avenues for the development of more effective and efficient predictive tools based on existing questionnaires combined with AI tools.

## Materials and Methods

### Participants.

A total of 600 participants were recruited across two studies through the online recruitment platform Prolific (Study 1, *N* = 200; Study 2, *N* = 400; Table 1). All participants provided written informed consent, and the study protocols were approved by the Yale University institutional review board (protocol #2000028824). Both studies involved collecting measures at initial and follow-up sessions after three weeks. At the initial session, participants first completed a standard questionnaire to assess depressive symptoms. They performed an established risk-taking task with happiness ratings (31, 40, 41) and provided written responses to nine free-response questions about their experiences (i.e., mood, motivation, sleep) over the past two weeks. Participants were invited to a follow-up study after three weeks and were asked to complete the standard questionnaire of depressive symptoms again. Approximately 78% of the initial participants completed both baseline and follow-up sessions (Study 1, final *N* = 179; Study 2, final *N* = 288), leaving a total of 467 from both studies with follow-up data.

**Table 1.** Participant demographics and depression scores in Study 1 and Study 2

	Study 1	Study 2	*Wilcoxon* test
*Recruitment*			
Initial I recruitment	200	400	–
Initial II retention (%)	–	324 (81)	–
Follow-up retention (%)	179 (90)	288 (72)	–
*Demographics*			
Mean age (SD)	42.5 (14.0)	43.5 (14.7)	*P* = 0.50
UK residence participants (%)	173 (97)	286 (99)	–
*Gender, No. (%)*			
Female	93 (52)	144 (50)	–
Male	85 (47)	139 (48)	–
Nonbinary	1 (1)	3 (1)	–
Prefer not to say	0 (0)	2 (1)	–
*Education level, No. (%)*			
Less than high school	1 (1)	4 (1)	–
High school	23 (13)	48 (17)	–
GED or equivalent	7 (4)	12 (4)	–
Associate degree	17 (9)	25 (9)	–
Some college no degree	18 (10)	65 (23)	–
Bachelor’s degree	74 (41)	87 (30)	–
Master’s degree	31 (17)	32 (11)	–
Professional degree	6 (3)	5 (2)	–
Doctoral degree	2 (1)	9 (3)	–
Prefer not to say	0 (0)	1 (0)	–
*Symptom measures (PHQ-9), mean (SD)*			
Initial depression symptoms	5.91 (5.21)	6.48 (5.35)	*P* = 0.21
Follow-up depression symptoms	5.81 (4.92)	6.80 (5.72)	*P* = 0.15
Depression symptoms change	−0.09 (3.39)	0.32 (3.25)	*P* = 0.20

We assessed whether initial depression severity was associated with attrition rates in either study. In both studies, we found that initial depression severity was not different between the participants who completed the study (Study 1, *N* = 179; Study 2, *N* = 288) and dropped out (Study 1, *N* = 20; Study 2, *N* = 109). For the 2-session Study 1, there was no difference between initial depression scores for participants who completed the study (mean PHQ-9 = 5.91, SD PHQ-9 = 5.21, median PHQ-9 = 5) and those who dropped out (mean PHQ-9 = 6.75, SD PHQ-9 = 6.30, median PHQ-9 = 5; Wilcoxon rank-sum test, *W* = 1816, *P* = 0.772). For the 3-session Study 2, there was no difference in initial depression scores between participants who completed the study (mean PHQ-9 = 6.48, SD PHQ-9 = 5.35, median PHQ-9 = 5) and those who dropped out (mean PHQ-9 = 6.90, SD PHQ-9 = 5.43, median PHQ-9 = 6; *W* = 16479, *P* = 0.442; *SI Appendix*, Fig. S7). Greater attrition was expected in Study 2 as it involved one additional session compared to Study 1 (see Experiment procedure below for details), and we increased our sample size accordingly. Although attrition reduced our statistical power, we found robust results across both studies.

### Data Inclusion.

All participants’ data were included in the data analyses if they completed both baseline and follow-up. No participants were excluded.

### Procedure.

#### Experiment procedure.

In both studies, we used the same experimental procedure, with one key modification in the initial data collection for Study 2. In Study 2, we divided the initial data collection into two shorter sessions. During the first session, participants completed a standard depression questionnaire and provided happiness ratings during a decision-making task. The second session took place a day after the first, during which participants provided written responses to nine neutral open-ended questions about their experiences. This division allowed us to investigate whether language sentiment could still predict changes in depressive symptoms, even when the written responses and initial depression scores were collected on separate days. In contrast, all baseline data, including depression scores, mood ratings during a decision task, and written responses, were collected on the same day in Study 1 (*SI Appendix,* Fig. S1*A* and Table S1*A*).

#### Standard depression questionnaire (PHQ-9).

PHQ-9 comprises nine questions that assess various depressive symptoms such as depressed mood, anhedonia, and insomnia/hypersomnia (18) (*SI Appendix*, Table S1*B*). Participants responded to each question on a 0 to 3 Likert scale based on the frequency of how many days they have experienced the corresponding symptom in the past two weeks (0 – not at all to 3 – nearly every day). We collected the PHQ-9 questionnaire twice, first at an initial session and second at a follow-up session after three weeks (*SI Appendix,* Fig. S8).

### Mood Experiences During Decision-Making.

#### Risky decision task.

This task presented a safe and a risky option in each trial (*SI Appendix,* Fig. S1*B*). The participants were asked to choose either option with the goal of maximizing their total points. All safe options earned 0 points. All risky options were mixed gambles with a potential gain and a potential loss, and the chance of winning or losing each risky option was fixed at 50%. When the risky option was chosen, the outcome was revealed. The amount of points earned or lost after each trial was reflected in the total points presented at the bottom of the task screen. After every 5 decision trials, the participants rated their current mood by answering the question, “How happy are you at the moment?” using a cursor on a line scaled from 0 to 100 (*SI Appendix,* Fig. S1*C*). Participants completed 100 decision trials and 20 mood ratings.

#### Computational modeling of sequential happiness ratings.

We used an established computational model of sequential happiness ratings during risky decision-making to dissect affective processes associated with language use in depression (31, 39–41). The model includes three terms that reflect the influence of different event types on current happiness. Certain rewards (CRs) mean the chosen safe rewards, expected values (EVs) represent the average return of the chosen gambles, and reward prediction errors (RPE) are the difference between the gamble outcome and gamble EVs:

[1] $$\begin{array}{c} Happiness\left(t\right)=w_{0}+w_{1}\sum_{j=1}^{t}\gamma^{t-j}CR_{j}\\ \;+w_{2}\sum_{j=1}^{t}\gamma^{t-j}EV_{j}+w_{3}\sum_{j=1}^{t}\gamma^{t-j}RPE_{j}, \end{array}$$

where t is the number of the current mood rating trial, j is a trial number, $w_{0}$ is a baseline mood parameter, other weights represent the degree to which event type modulates mood ratings, and *γ* is a forgetting factor bounded from 0 to 1, allowing more recent events to be weighted greater than earlier events. For this task in which all CRs were zero, the equation simplifies to:

[2] $$Happiness(t)=w_{0}+w_{1}\sum_{j=1}^{t}\gamma^{t-j}EV_{j}+w_{2}\sum_{j=1}^{t}\gamma^{t-j}RPE_{j},$$

We focused on the happiness baseline mood parameter $w_{0}$ that conceptually captures baseline happiness setpoints and tested for associations with depressive symptom scores and language measures.

### Language Describing Past Experiences.

#### Depression-related open-ended questions in an emotionally neutral tone.

We developed nine neutral, open-ended questions that ask about past experiences known to differ in people with depression (Table 2 and *SI Appendix,* Table S1*D*). All questions were rephrased from the original nine questions in the PHQ-9. It is noteworthy that these questions also correspond to the criteria in DSM-5 for diagnosing Major Depressive Disorder. The questions aim to draw out information about participants’ unique past experiences related to 1) mood, 2) interest levels, 3) eating habits, 4) sleep patterns, 5) physical activity, 6) energy levels, 7) self-perceptions, 8) concentration, and 9) overall life attitude including both satisfaction and dissatisfaction. By doing so, we aimed to construct a language corpus that captures the variability in past experiences among individuals with varying levels of depression.

**Table 2.** Standard depression questionnaire (PHQ-9; left column) and modified depression-related experience questions (right column)

PHQ-9 question categories (“Over the last 2 weeks, how often have you been bothered by any of the following problems?”)	Depression-related experience questions
Feeling down, depressed, or hopeless	Could you describe your general mood in the past 2 weeks? Has your mood been higher or lower than usual? Are there any particular emotions that you have been feeling a lot lately?
Little interest or pleasure in doing things	Take a moment to think about things you usually enjoy. How would you describe your level of interest in these things in the past 2 weeks? Has it been higher or lower than usual?
Poor appetite or overeating	How have you been eating in the past 2 weeks? Is there anything that has been different compared to usual (e.g., eating more or less than usual)?
Trouble falling or staying asleep, or sleeping too much	How would you describe your sleep patterns lately? Is there anything that has been different compared to usual?
Moving or speaking so slowly that other people could have noticed. Or the opposite—being so fidgety or restless that you have been moving around a lot more than usual	In the past 2 weeks, has sitting still, moving, or talking been harder or easier than usual?
Feeling tired or having little energy	Sometimes we feel tired and exhausted, and sometimes we feel full of energy. How would you describe your energy level in the past 2 weeks?
Feeling bad about yourself—or that you are a failure or have let yourself or your family down	How have you been feeling about yourself in the past 2 weeks?
Trouble concentrating on things, such as reading the newspaper or watching television	How would you describe your thinking, concentration, and decision-making in the past 2 weeks? Is there anything that has been harder or easier than usual?
Thoughts that you would be better off dead, or of hurting yourself	Think about your life overall. Is there anything that you are particularly satisfied or dissatisfied with?

However, in contrast to the original PHQ-9 questions, we framed the questions to let participants freely narrate their past experiences in their own language. For example, the depressed mood question in PHQ-9, “Over the last 2 weeks, how often have you been bothered by feeling down, depressed, or hopeless?” was correspondent to “Could you describe your general mood in the past 2 weeks?” This framing of the questions allowed participant responses concerning depression symptom-related experiences (i.e., mood, sleep) and self-evaluation of those experiences to be either positive, negative, or both. Given similar experiences, two hypothetical respondents might describe their experiences differently in written language, providing insights into their current and future depressive symptoms.

The questions additionally inquired about the participants’ experiences in comparison to their usual lives (e.g., “How would you describe your sleep patterns lately? Is there anything that has been different compared to usual?”). This comparative framing was added to be consistent with one of the main diagnostic criteria for Major Depressive Disorder. Under DSM-5, it is crucial to identify whether the individual description of depressive symptoms is atypical from their usual experiences (e.g., slept a lot less or more than usual). In alignment with this diagnostic focus, we included a comparative question to gather information about the extent to which their past two-week experiences deviated from their usual lives, as reflected in their written language.

Participants were instructed to freely respond while avoiding disclosing any identifiable information. This approach enabled us to gather extensive written responses detailing participant past depressive symptom-related experiences, all within a time frame of less than 10 min on average (Study 1, mean = 8.14 mins, SD = 5.14 mins; Study 2, mean = 9.78 mins, SD = 6.40 mins). Per prompt, participants wrote a mean of 22.44 words (SD = 14.35) in Study 1 and 25.23 words (SD = 15.51) in Study 2 (Table 3).

**Table 3.** Descriptive statistics of text response word counts in Study 1 and Study 2

Study 1					
Question number	Mean	SD	Median	Min	Max
1	28.91	17.70	25	3	126
2	22.59	13.46	19	1	83
3	20.00	11.84	17	1	76
4	22.71	12.50	20	2	75
5	17.44	10.46	15	1	81
6	19.96	10.39	19	1	68
7	20.42	13.68	17	1	91
8	21.09	12.39	18	1	72
9	28.86	26.69	23	1	264
Mean of each question	22.44	14.35	19.22	1.33	104
Mean of total	201.98	95.62	178	30	638
Study 2					
Question number	Mean	SD	Median	Min	Max
1	31.79	18.34	28	1	136
2	26.36	15.98	23	1	112
3	23.91	13.92	21	2	102
4	24.48	13.81	22	1	110
5	19.62	12.57	16	1	94
6	22.30	11.80	20	1	76
7	24.07	16.21	20	1	145
8	23.62	13.90	20	1	99
9	30.93	23.02	25	1	203
Mean of each question	25.23	15.51	21.67	1.11	119.67
Mean of total	227.09	108.27	205	32	858

#### Manual sentiment analysis by human raters.

We recruited an additional 470 human raters who were fluent English speakers through the same online recruitment platform used for collecting the original responses (female *N* = 234, male *N* = 236; mean age = 38.3, SD age = 13.3). To maintain independence, the original participants who wrote the texts in our main studies were not eligible to participate in the rating study. Each of the 470 human raters was assigned three randomly selected text responses from different participants for each of the nine open-ended questions. This resulted in each rater giving one positive and one negative sentiment rating for each of the 27 text responses. All human raters were instructed to rate the positivity and negativity of each text on a scale of 0 to 10 (*SI Appendix,* Table S7). The instructions on rating sentiment were similar to those provided to ChatGPT (Table 4). Using this approach, we confirmed that 167 (93%) and 276 (96%) of participants’ responses from Study 1 and Study 2, respectively, received sentiment ratings from at least two human raters for all nine responses. At the response level, only 1% of all text responses from both studies (12 out of 1,611 texts in Study 1; 13 out of 2,592 texts in Study 2) were not rated twice.

**Table 4.** ChatGPT prompt (left column) and human rating prompt (right column)

ChatGPT rating prompt	Human rating prompt
“Rate the sentiment of the text. How negative and positive is this text on a 0 to 10 scale? Answer always only with two numbers separated by a comma. First number 0 being ‘not negative at all’ to 10 being ‘very negative’, and second number 0 being ‘not positive at all’ to 10 being ‘very positive.’ Please make sure you don’t print out any letters. Here is the text:”	“In the study, you will be asked to rate the sentiment of the texts. Prior to this study, real human participants were asked to write about their experiences. Your role is to rate the sentiment of those texts—how negative and positive is each text on a 0 to 10 scale? Negativity score 0 means not negative at all, and 10 being very negative. Positivity score 0 means not positive at all, and 10 being very positive. This study will present you with 27 texts in total. To proceed to the next text, please make sure to give both positivity and negativity scores for each text.”

We assessed consistency among human raters and found a strong interrater group correlation between average sentiment scores for first compared to second raters in both Study 1 (Pearson *R* = 0.88, *P* < 0.001) and Study 2 (*R* = 0.91, *P* < 0.001; *SI Appendix,* Fig. S9 and Table S8). Given that humans may reach reasonably different conclusions about responses, we took an unbiased approach and averaged across first and second raters. We used the first rating for those texts missing the second rating (1% for both studies). Even when we excluded these texts with only one response and ran separate analyses for scores based on only the first or second ratings, all prediction results were unchanged (*SI Appendix,* Fig. S10).

In our main analyses, we used a composite sentiment difference score by subtracting the mean negative sentiment rating from the mean positive sentiment rating for each response and averaging them across nine responses. We also replicated all our findings using the positive and negative sentiment scores separately (*SI Appendix,* Table S2).

#### Automatic sentiment analyses using LIWC and ChatGPT.

We used two additional automatic analyses of language sentiment: LIWC 2022 [version 1.8.0; LIWC (20, 22)] and Generative Pre-trained Transformer language model implemented in AI chatbot, ChatGPT. LIWC quantifies individual emotional word count score by computing how many emotional words are included in a given text as a fraction of the total number of words (48). We used the built-in dictionary of LIWC and selected the positive and negative tone categories in the dictionary (abbreviations: tone_pos and tone_neg, respectively). To count the overall emotional word use, we computed the average score of each of the positive and negative emotional words from the nine written responses. The final emotional word count score for each participant was calculated by taking the difference between the mean positive tone score and the mean negative tone score. For instance, a participant with an affective tone word count score of 3.0 indicated that they included 3% more positive sentiment words compared to negative sentiment words in their responses on average. Given that LIWC scores are computed based on the number of total words and considering the brief nature of our collected text responses (Table 3), we addressed the potential undue influence of total word counts by taking two additional approaches. First, we applied data exclusion based on word counts and recomputed the LIWC sentiment scores. Second, we used block-level LIWC sentiment scores by concatenating the nine responses into a single block of text per participant (*SI Appendix* *f*or details). The analyses involving LIWC sentiment scores were then reperformed using these modified scores.

ChatGPT is an AI chatbot that uses generative language models trained on large language corpora developed by OpenAI. Recent research has demonstrated the sentiment scoring capabilities of ChatGPT (24). In this study, we used the GPT versions 3.5 and 4 (*SI Appendix,* Figs. S3 and S4) to assess the sentiment of written responses provided by online participants. To compute an overall sentiment score for each participant's set of nine written responses, we averaged the sentiment scores from the nine texts. Consistent with LIWC sentiment scores, the final sentiment score was calculated as the difference between the mean positive and negative sentiment scores, quantifying the relative use of positive emotional tone compared to negative emotional tone. We also tested our hypotheses separately using positive and negative sentiment scores from human raters, ChatGPT, and LIWC (*SI Appendix*, Table S2).

### Statistical Analysis.

All analyses were conducted in MATLAB (version R2020a) and R (version 4.2.3). Our main hypothesis involved testing the prediction of PHQ-9 scores, and we confirmed that the PHQ-9 scores at baseline and follow-up were possibly skewed (*SI Appendix,* Fig. S8). Thus, we used nonparametric tests for all analyses. A Spearman correlation coefficient *ρ* was computed for correlational analyses between variables under a nonlinear assumption. For prediction analyses, we used robust linear regression to mitigate the undue influence of outliers in the least square model fitting process (49). In the robust linear regression, we accounted for initial depression and important covariates (age, gender, education). All robust linear regression models were performed using the “lmrob” function in R with the default setting of “KS2014” (50). Significance of P-value was set at 0.05. We reported beta coefficients, SE, t-statistics, *P*-values, and Cohen’s $f^{2}$ values. We followed the established convention for interpretating the Cohen’s $f^{2}$ effect size values (i.e., $f^{2}\geq 0.02$ = small, $f^{2}\geq 0.15$ = medium, $f^{2}\geq 0.35$ = large) (51).

#### Leave-one-out cross-validation.

We conducted leave-one-out cross-validation within each study dataset to predict changes in depression scores while leaving out the predicted participant’s data during each iteration of the model fitting. We used the same robust linear regression model for the leave-one-out cross-validation and reported the root mean squared error (RMSE) and mean absolute error (MAE). The Pearson correlation coefficient was computed between the predicted and actual change values.

#### Model comparison.

To test whether the prediction model with sentiment scores yields better prediction performance, we conducted a model comparison with the nested model that excludes sentiment scores. An ANOVA using the Wald test was conducted to test the significance of the model difference.

## Supplementary Material

Appendix 01 (PDF)

## Data Availability

Analysis scripts and all experiment de-identified data have been deposited in GitHub (https://github.com/RutledgeLab/2024_language_sentiment_depression) (52). Raw text data from written responses are not publicly available due to their sensitive and personal nature.
