# Supplementary material for: Language sentiment predicts changes in depressive symptoms
Source: Proc Natl Acad Sci U S A. 2024 Sep 16;121(39):e2321321121. doi: 10.1073/pnas.2321321121 (PMC11441484; doi:10.1073/pnas.2321321121)
Supplement: Supplementary file 1 — Appendix 01 (PDF) [file pnas.2321321121.sapp.pdf]

PNAS

Supporting Information for

Language sentiment predicts changes in depressive symptoms.

Jihyun K. Hur, Joseph Heffner, Gloria W. Feng, Jutta Joormann, and Robb B. Rutledge

*Corresponding author: Robb B. Rutledge

Email: robb.rutledge@yale.edu

This PDF file includes:

Supporting texts 1 to 4
Figures S1 to S10
Tables S1 to S8

Supplementary Text 1: Calculating the Composite Sentiment Score

Upon collection of the written responses to the nine emotionally neutral, open-ended questions, we rated the positive and negative sentiment of each response per participant using manual (i.e., human raters) and automated (i.e., ChatGPT and Linguistic Inquiry and Word Count; LIWC) sentiment rating methods. We computed the mean positive and negative sentiment scores and used the difference score (e.g., mean positive minus mean negative sentiment scores) as a composite sentiment score (*SI Appendix,* Table S7).

Supplementary Text 2: Testing Prediction with Sentiment Ratings from Female versus Male Raters, or Older versus Younger Raters

We tested whether the demographics of human raters (*N* = 470) impacted both the sentiment ratings and the prediction results (female *N* = 234, male *N* = 236; mean age = 38.3, SD age = 13.3). We recalculated all participant average sentiment ratings by grouping human raters based on their sex at birth (female or male) and age (older or younger). The median rater age of 35 was used to divide the raters into age groups for both studies. These analyses lead to four average sentiment ratings computed for each participant, averaged ratings from female raters, male raters, older raters, and younger raters. We then compared the averaged sentiment ratings from female versus male raters and from older raters versus younger raters within participant, testing for any consistent differences in sentiment ratings between the demographic groups.

The average sentiment ratings from female raters were more positive than those from male raters in both Study 1 and Study 2 (Study 1, Wilcoxon signed-rank test, *V* = 9421.5, *P* = .049, *R* = .147; Study 2, Wilcoxon signed-rank test, *V* = 25860, *P* < .001, *R* = .218). Our main findings are unchanged when we analyzed ratings from either group separately to predict future depression beyond current symptom levels (Study 1, female raters β = -0.21, *SE* = 0.08, *t* = -2.81, *P* = .006, f_β^2 = 0.043, male raters β = -0.16, *SE* = 0.08, *t* = -1.97, *P* = .051, f_β^2 = 0.014; Study 2, female raters β = -0.13, *SE* = 0.06, *t* = -2.26, *P* = .025, f_β^2 = 0.009, male raters β = -0.23, *SE* = 0.06, *t* = -4.12, *P* < .0001, f_β^2 = 0.034). Similarly, we conducted an analysis based on age and discovered that older raters (age over 35) gave more positive sentiment ratings than younger raters (age up to 35) in both studies (Study 1, Wilcoxon signed-rank test, *V* = 10294, *P* = .001, *R* = .241; Study 2, Wilcoxon signed-rank test, *V* = 27916, *P* < .0001, *R* = .327). Despite these interesting differences, ratings from either age group were sufficient to independently predict changes in depression (Study 1, older raters β = -0.19, *SE* = 0.08, *t* = -2.43, *P* = .016, f_β^2 = 0.033, younger raters β = -0.26, *SE* = 0.08, *t* = -3.41, *P* < .001, f_β^2 = 0.060; Study 2, older raters β = -0.15, *SE* = 0.06, *t* = -2.47, *P* = .014, f_β^2 = 0.033, younger raters β = -0.19, *SE* = 0.06, *t* = -3.45, *P* < .001, f_β^2 = 0.030). Although demographic variables such as age and sex can influence

sentiment ratings, we find that sentiment ratings from all groups are able to predict depression trajectories.

Supplementary Text 3: Testing Prediction with the Linguistic Distance Measure

We investigated whether linguistic distance could predict changes in depression using the same text processing and computation methods as previous research[1]. Specifically, linguistic distance was computed by averaging LIWC-based social and temporal distance scores. Using LIWC-22, we calculated the social distance score as the proportion of non-first-person singular pronouns (i.e., you, we, she/he, they) relative to all pronouns used (i.e., I, you, we, she/he, they). The ‘Personal pronouns' categories in LIWC were used to compute social distance scores (i.e., I, you, we, she/he, they). The temporal distance score was calculated as the proportion of non-present-tense words relative to all tense words, using time orientation categories in LIWC (i.e., focuspast, focuspresent, focusfuture). While Nook et al. (2022) used the three verb tense categories (i.e., past, present, and future) originally included as the ‘Common verbs' subcategories of LIWC 2007, subsequent versions, including the one we used, substituted these tense categories with time orientation ones that include not only verbs (e.g., asks) but also general time expressions (e.g., ongoing).

In both studies, linguistic distance did not predict changes in depression after three weeks (Study 1, $\beta_{Linguistic\ distance}$ = 2.74, *SE* = 2.22, *t* = 1.23, *P* = .220, $f^2_{\beta_{Linguistic\ distance}}$= 0.012; Study 2, $\beta_{Linguistic\ distance}$ = -0.14, *SE* = 2.20, *t* = -0.06, *P* = .949, $f^2_{\beta_{Linguistic\ distance}}$= -0.002; see *SI Appendix,* Fig. S2*A*). Considering the inherently self-referential nature of the nine open-ended questions, we did not predict that the choice to use such language would be predictive of changes in depression in our dataset. Given the correlation between linguistic distance and sentiment scores with current depression, we conducted tests to determine if the predictive power of sentiment scores was reduced after accounting for the linguistic distance measure. Our findings indicate that sentiment scores continued to robustly predict changes in depression even after controlling for the linguistic distance measure and initial depression scores (Study 1, $\beta_{Human\ raters}$ = -0.27, *SE* = 0.09, *t* = -3.13, *P* = .002, $f^2_{\beta_{Human\ raters}}$= 0.046; Study 2, $\beta_{Human\ raters}$ = -0.20, *SE* = 0.06, *t* = -3.14, *P* = .002, $f^2_{\beta_{Human\ raters}}$= 0.021; see *SI Appendix,* Fig. S2*B*).

Supplementary Text 4: Testing Robustness of LIWC-Based Sentiment Prediction of Future Depression Trajectories

As LIWC computes the percentages of words (i.e., sentiment) included in each text, all LIWC linguistic scores are affected not only by the number of target words but also by the total number of words. Within the context of brief written responses, the data from such computation require careful interpretation, given that a very low total word count may lead to a large variance in the scores with minimal differences in the number of the target words (e.g., 1 negative tone word out of 2 total words = 50% negative score vs. 1 negative tone word out of 10 total words = 10% negative score).

In this study, LIWC sentiment scores were computed per response, which included 22.44 and 25.23 words on average (Table 3 in the main text). In our main analyses, we found that LIWC sentiment scores did not predict changes in depression (Fig. 2*B* in the main text), even when we confirmed a reliable and strong correlation between LIWC sentiment scores and current depression across the two studies (Study 1, ρ = -.52, *P* < .001; Study 2, ρ = -.47, *P* < .001). However, to address the psychometric concern, we performed two additional LIWC analyses by 1) excluding responses and participants with low word counts and 2) employing block-level LIWC sentiment scores computed from a concatenated block of text responses. Using these approaches, we re-investigated whether LIWC sentiment scores could predict future depression even after accounting for initial depression scores.

First, we excluded all responses that had fewer than 10 words in our analyses and recomputed the average LIWC sentiment scores. To take a more conservative approach, we additionally excluded participants who wrote fewer than 10 words for more than five responses (Study 1, response $N_{Excluded}$= 152, participant $N_{Excluded}$= 9; Study 2, response $N_{Excluded}$= 189, participant $N_{Excluded}$= 9). Even after the data exclusion based on word counts, we found that LIWC sentiment scores did not predict changes in depression (Study 1, β_{LIWC}= -0.06, *SE* = 0.08, *t* = -0.73, *P* = .464, $f^2_{\beta_{LIWC}}$ = 0.003; Study 2, β_{LIWC} = -0.01, *SE* = 0.06, *t* = -0.21, *P* = .836, $f^2_{\beta_{LIWC}}$ = -0.002; *SI Appendix*, Table S4). Human and ChatGPT sentiment scores still predicted changes in depression even after the data exclusion.

However, using a cutoff word count of 10 may still not be sufficient to address this concern. In the second additional analysis, we aggregated all nine texts into one block and re-applied LIWC analysis to compute one sentiment score per participant. Since the total words of the concatenated nine texts was 201.98 and 227.09 on average in Study 1 and Study 2, respectively, we expected that this undue influence by the number of total words on the sentiment scores would be reduced using the block-level scores. We confirmed a positive correlation between the average and block-level sentiment scores (Study 1, *R* = .90, *P* < .001; Study 2, *R* = .93, *P* < .001). Two participants from each study whose concatenated texts had fewer than 50 words were excluded. We found that block-level LIWC sentiment scores were negatively

associated with current depression (Study 1, ρ = -.48, P < .001; Study 2, ρ = -.44, P < .001), consistent with the average LIWC sentiment scores. However, in the robust linear regression model, block-level LIWC sentiment did not predict changes in depression in either study (Study 1, $\beta_{Block-level\ LIWC}$ = -0.02, *SE* = 0.08, *t* = -0.28, *P* = .780, $f^2_{\beta_{Block-level\ LIWC}}$ = 0.002; Study 2, $\beta_{Block-level\ LIWC}$ = 0.01, *SE* = 0.06, *t* = 0.12, *P* = .904, $f^2_{\beta_{Block-level\ LIWC}}$ = -0.002; *SI Appendix*, Fig. S5). Results from LIWC sentiment scores showed that excluding data based on word counts or using block-level analysis did not impact the lack of predictive ability of LIWC for depression trajectories in the context of this study.

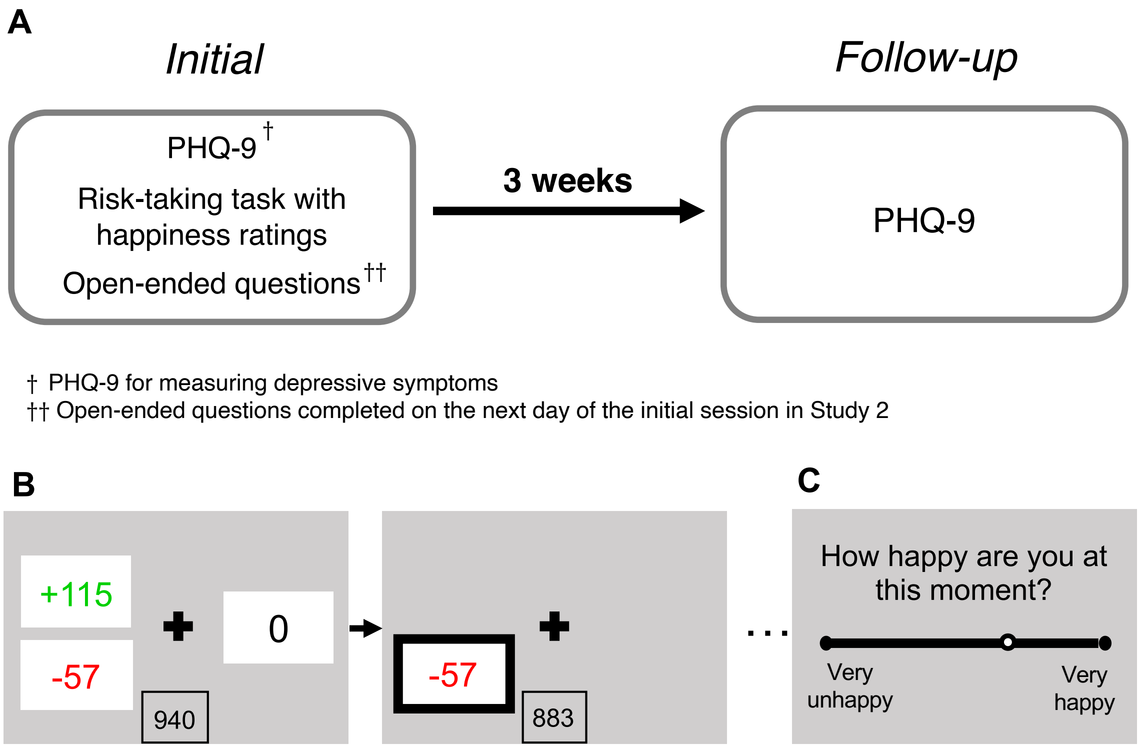

Figure S1. Study procedure and decision-making task. (A) Participants completed initial and follow-up sessions. Standard measures of depression and anxiety, happiness ratings during a risk-taking task, and written responses to nine brief open-ended questions were collected at the initial session. At follow-up after three weeks, the standard depression and anxiety questionnaires were completed again. (B) Decision-making trials with a risky option (left) and a safe option (right). When a participant chooses a risky option (e.g., winning 115 points for a 50% chance or losing 57 points for a 50% chance), the outcome is revealed on the next screen (e.g., lost 57 points). The cumulative total points are always presented at the bottom of the screen. (C) Happiness rating trial. Participants rated happiness every 5 trials during the task and this was converted to 0 to 100.

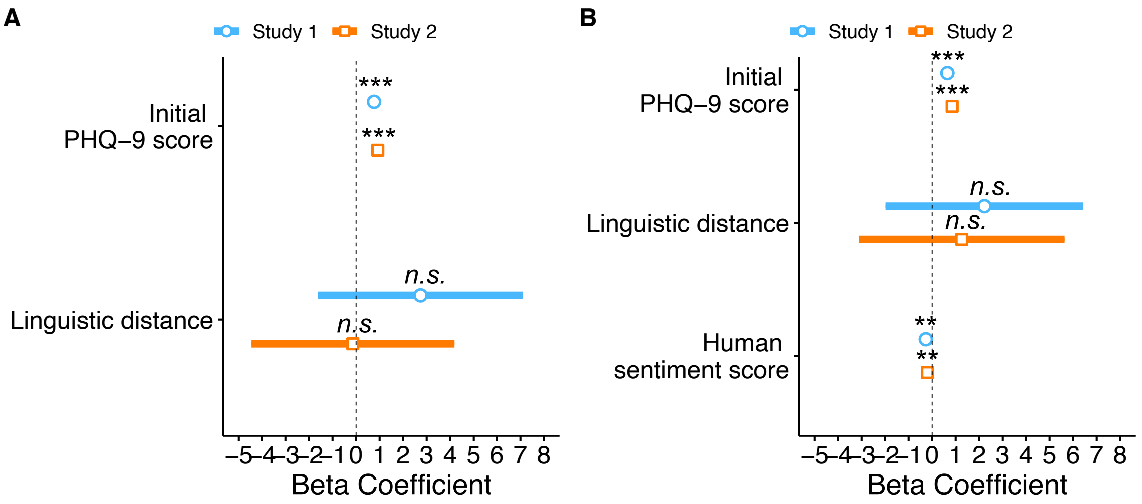

Figure S2. Language sentiment measure predicted future depression even after controlling for linguistic distance. (A-B) Robust linear regression of future depressive symptom scores (PHQ-9) after three weeks using (A) linguistic distance only and (B) using both linguistic distance and human sentiment ratings. Note that in all models, the PHQ-9 score at follow-up was used as a dependent variable, and age, gender, and education level were included as covariates. (A) Linguistic distance did not predict changes in depression scores after three weeks. (B) In analyses using both linguistic distance and human sentiment scores, more negative sentiment scores predicted increased depressive symptoms at three-week follow-up in both studies, even after controlling for linguistic distance and initial depression scores. ** P < .05; ** P < .01; *** P < .001*

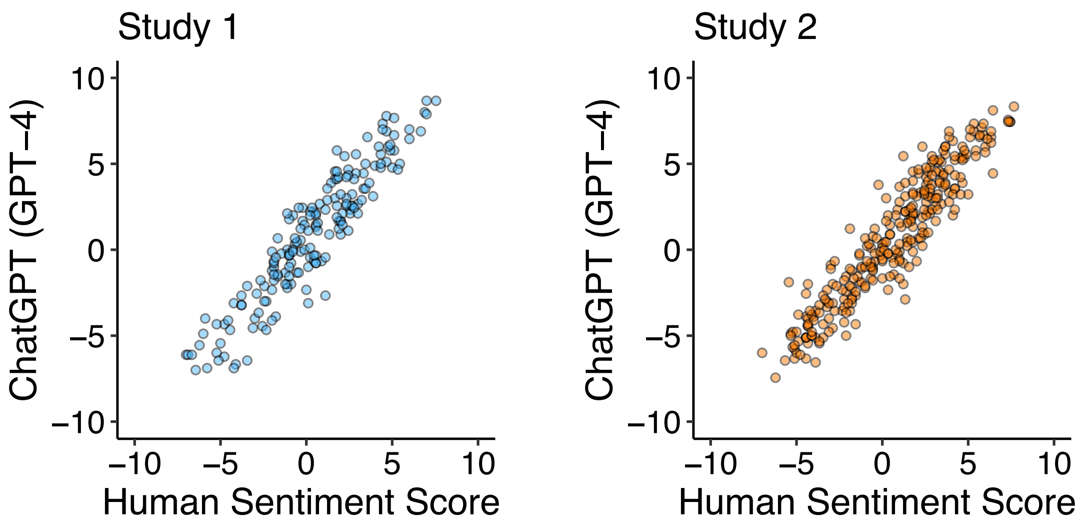

Figure S3. ChatGPT (GPT-4) ratings are correlated with human sentiment ratings. Spearman coefficients ρ between LLM-based automated sentiment analysis with ChatGPT (GPT-4) and human ratings for Study 1 (ρ = .96, $P < .001$) and Study 2 (ρ = .96, $P < .001$).

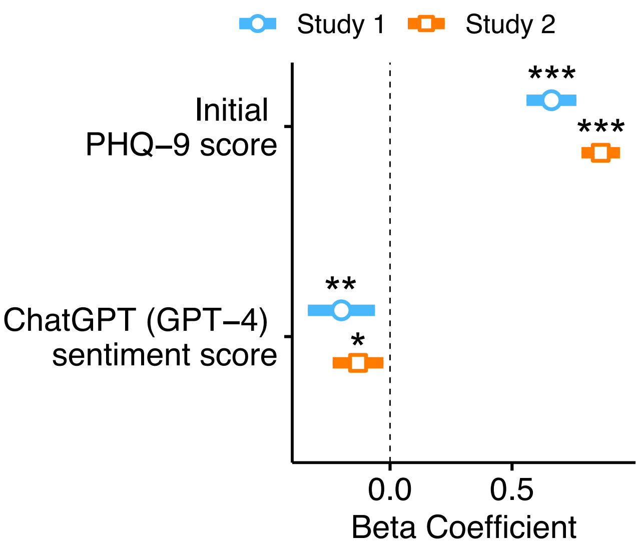

Figure S4. ChatGPT (GPT-4) sentiment ratings predict future depression. Robust linear regression predicted future depressive symptom scores (PHQ-9) after three weeks using ChatGPT (GPT-4) sentiment ratings. Note that in the model, the PHQ-9 scores at follow-up were used as a dependent variable, and age, gender, and education level were included as covariates. ChatGPT (GPT-4) sentiment ratings predicted changes in depression (Study 1, $\beta_{ChatGPT\ (GPT-4)}$= -0.20, *P* = .005; Study 2, $\beta_{ChatGPT\ (GPT-4)}$= -0.13, *P* = .014) * *P < .05;* ** *P < .01;* *** *P < .001*

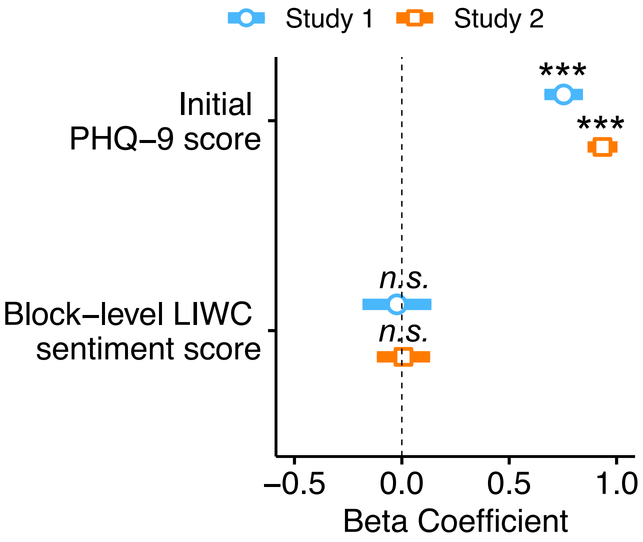

Figure S5. Block-level LIWC sentiment scores did not predict future depression. Robust linear regression predicted future depressive symptom scores (PHQ-9) after three weeks using block-level LIWC sentiment ratings. Block-level means that all nine responses were concatenated in a block per participant before applying LIWC analysis and computing LIWC sentiment score. To ensure reliable computation of LIWC analysis, two participants from each study whose concatenated text responses included fewer than 50 words were excluded from this analysis. PHQ-9 scores at follow-up were used as a dependent variable, and age, gender, and education level were included as covariates. Block-level LIWC sentiment ratings did not predict changes in depression. ** P < .05; ** P < .01; *** P < .001*

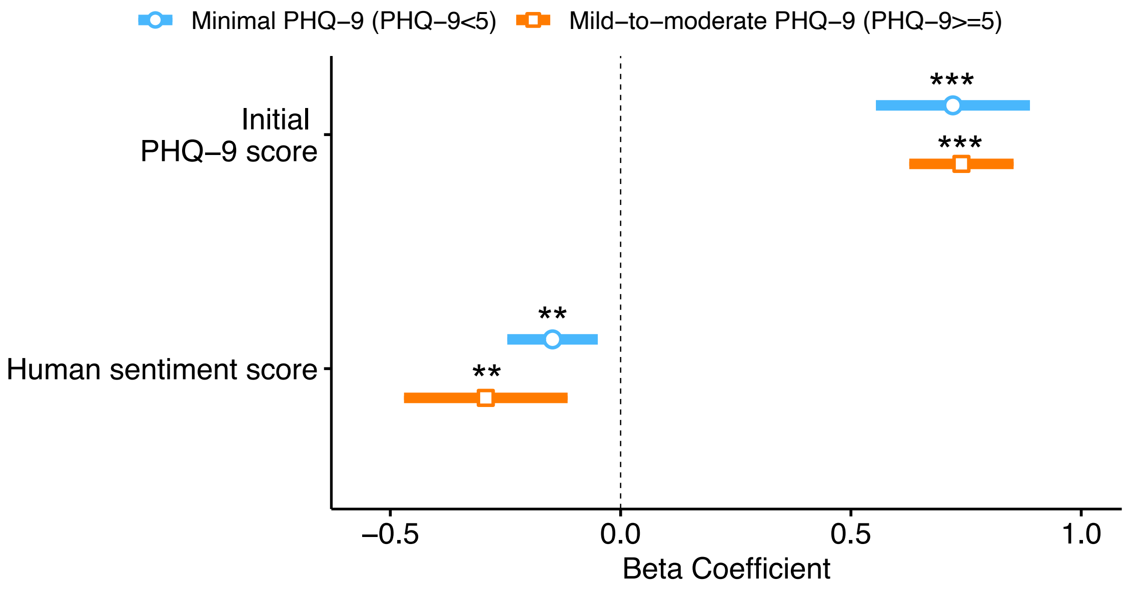

Figure S6. Language sentiment predicted changes in depression for both minimal and mild-to-moderate levels of depression. Robust mixed effect linear regression of future depression scores after three weeks using participants with minimal levels of depression (i.e., PHQ-9 less than 5) and mild-to-moderate levels of depression (i.e., PHQ-9 of 5 or greater than 5). To test if the sentiment prediction was consistent for different levels of initial depressive symptoms, we used a PHQ-9 cutoff of 5, which grouped participants into two groups with similar sizes (Study 1, minimal PHQ-9 $N = 104$, mild-to-moderate PHQ-9 $N = 75$; Study 2, minimal PHQ-9 $N = 147$, mild-to-moderate PHQ-9 $N = 141$). To address the reduced statistical power due to smaller participant numbers, data from Studies 1 and 2 were combined, and the study identifiers were accounted for as a random intercept in each robust mixed linear model. Human sentiment scores still predicted future depression above and beyond initial depression scores for both minimal levels of depression and mild-to-moderate levels of depression. * $P < .05$; ** $P < .01$; *** $P < .001$

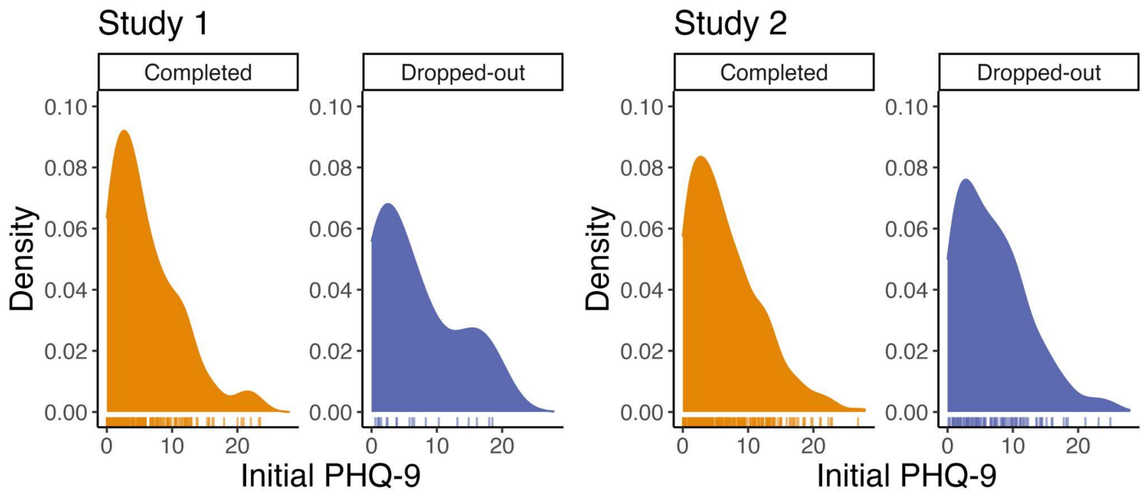

Figure S7. PHQ-9 distributions for the participants who completed or dropped out of the study. The x-axis shows initial PHQ-9 scores, indexing depression severity, while the y-axis shows the estimated density. Orange indicates participants who completed the study, while purple indicates those who dropped out after completing the initial experiment and did not return for one subsequent session in Study 1 (the three-week follow-up) or two subsequent sessions in Study 2 (the second baseline session and three-week follow-up session). The rug plots at the base of each density curve represent individual participant scores.

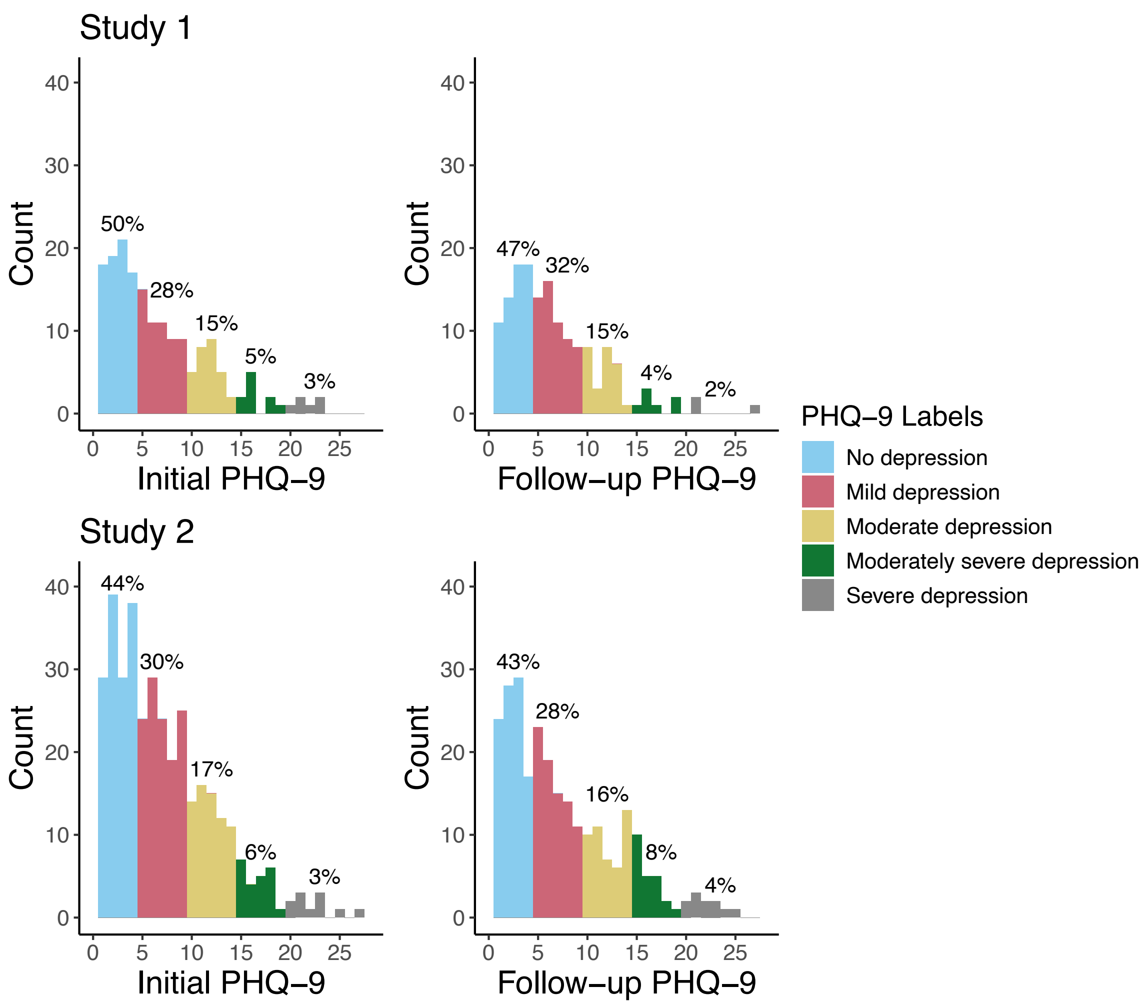

Figure S8. Distributions of PHQ-9 scores at baseline and follow-up. The x-axis shows PHQ-9 scores quantifying depression symptom severity, and the y-axis shows participant counts using a bin size of 1. Bar colors refer to PHQ-9 labels suggested in the literature, and the proportion of participants for each label is shown above each label.

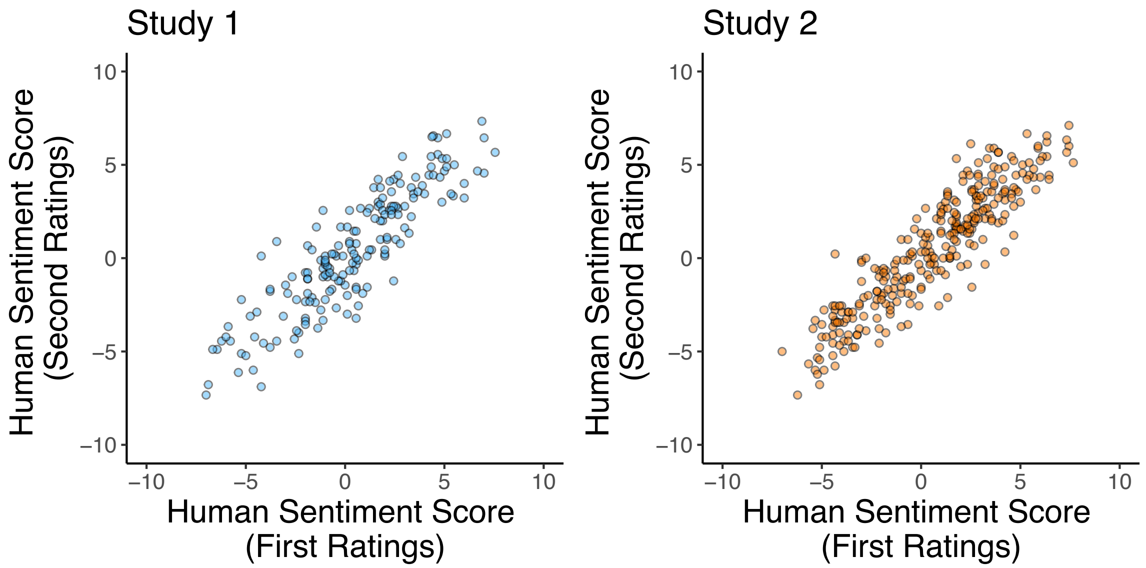

Figure S9. Correlations between the first and second sentiment ratings from two different groups of nine human raters. The x-axis represents the human-rated sentiment scores from the first group, while the y-axis represents the scores from the second group. Each dot indicates an individual participant's sentiment score, based on average ratings across all nine free responses. Responses that did not receive at least two ratings were excluded from the analysis [Study 1 response $N_{Excluded}$ = 12 (1%); Study 2 response $N_{Excluded}$ = 13 (1%)]. High correlations in both Study 1 and Study 2 support the reliability between two groups of raters.

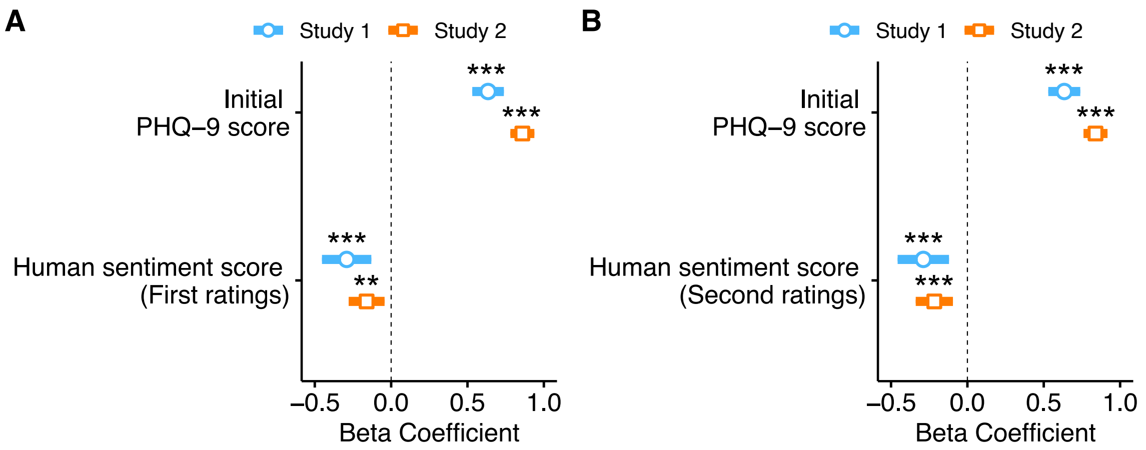

Figure S10. Language sentiment from both first- and second-rater groups predicted changes in depression. Robust linear regression was used to predict future depressive symptom scores (PHQ-9) after three weeks based on human-rated sentiment ratings by (A) the first group of nine raters and (B) the second group of nine raters. The sentiment of nine text responses from each participant was rated by different human raters, and the average sentiment scores were used as the primary predictor of future depressive symptoms. PHQ-9 scores at follow-up were used as the dependent variable, while age, gender, and education level were included as covariates. A small proportion of text responses (1% for each study) was excluded from this analysis to ensure that an identical list of text responses was used for computing the average first and second sentiment ratings. ** P < .05; ** P < .01; *** P < .001*

Table S1. Survey questions and order.

A. All surveys and order.

Session	Survey order	Survey
**Initial**	1	Patient Health Questionnaire-9 (PHQ-9)
	2	Online participation and demographic information questionnaire
	3	Depression-related open-ended questions in an emotionally neutral tone
**Follow-up**	1	PHQ-9

B. PHQ-9 question order, wording, and scoring.

Question order	Question wording	Likert scale
**1**	Over the last 2 weeks, how often have you been bothered by any of the following problems?<br>Little interest or pleasure in doing things	0 (not at all) – 3 (nearly every day)
**2**	… Feeling down, depressed, or hopeless	0 - 3
**3**	… Trouble falling or staying asleep, or sleeping too much	0 - 3
**4**	… Feeling tired or having little energy	0 - 3
**5**	… Poor appetite or overeating	0 - 3
**6**	… Feeling bad about yourself – or that you are a failure or have let yourself or your family down	0 - 3
**7**	… Trouble concentrating on things, such as reading the newspaper or watching television	0 - 3
**8**	… Moving or speaking so slowly that other people could have noticed. Or the opposite – being so fidgety or restless that you have been moving around a lot more than usual	0 - 3
**9**	… Thoughts that you would be better off dead, or of hurting yourself	0 - 3

C. Online participation and demographic information questionnaire order, wording, and scoring.

Question order	Question wording	Response type
**1**	How many submissions have you completed on Prolific so far (including this one)?	1 (this is my first time)<br>2-10<br>11-50<br>51-100<br>101+<br>Prefer not to say

**2**	How many hours per week do you do studies on Prolific?	Less than 1 hour<br>1-2 hours<br>2-4 hours<br>4-8 hours<br>8-20 hours<br>More than 20 hours<br>Prefer not to say
**3**	Do you do online experiments on other platforms (e.g., MTurk, Qualtrics)?	Yes<br>No<br>Prefer not to say
**4**	Have you ever been diagnosed with major depression?	Yes<br>No<br>Prefer not to say
**5**	Have you ever been diagnosed with an anxiety disorder?	Yes<br>No<br>Prefer not to say
**6**	Are you currently taking a SSRI drug (like citalopram/Celexa, fluoxetine/Prozac, paroxetine/Paxil, escitalopram/Lexapro, sertraline/Zoloft)?	Yes<br>No<br>Prefer not to say
**7**	Are you currently taking a SNRI drug (like venlafaxine/Effexor, duloxetine/cymbalta)?	Yes<br>No<br>Prefer not to say
**8**	Are you currently taking a NDRI drug (like buproprion/Wellbutrin/Zyban, methylphenidate/Ritalin)?	Yes<br>No<br>Prefer not to say
**9**	Year of birth	[<1944 1944 1945 … 2002 2003 >2003]<br>Prefer not to say
**10**	Gender	Female<br>Male<br>Prefer not to say
**11**	Overall, how satisfied are you with your life nowadays?	0 (not at all) – 10 (very)
**12**	Highest qualification achieved	No high school diploma<br>High school graduate<br>GED or equivalent<br>Some college (no degree)<br>Associate degree<br>Bachelor’s degree<br>Master’s degree<br>Professional school degree<br>Doctoral degree<br>Unknown<br>Prefer not to say

D. Depression-related open-ended questions in an emotionally neutral tone order, wording, and scoring.

Question order	Question wording
**1**	Could you describe your general mood in the past 2 weeks? Has your mood been higher or lower than usual? Are there any particular emotions that you have been feeling a lot lately?
**2**	How would you describe your level of interest in these things in the past 2 weeks? Has it been higher or lower than usual?
**3**	How have you been eating in the past 2 weeks? Is there anything that has been different compared to usual (e.g., eating more or less than usual)?
**4**	How would you describe your sleep patterns lately? Is there anything that has been different compared to usual?
**5**	In the past 2 weeks, has sitting still, moving, or talking been harder or easier than usual?
**6**	Sometimes we feel tired and exhausted, and sometimes we feel full of energy. How would you describe your energy level in the past 2 weeks?
**7**	How have you been feeling about yourself in the past 2 weeks?
**8**	How would you describe your thinking, concentration, and decision making in the past 2 weeks? Is there anything that has been harder or easier than usual?
**9**	Think about your life overall. Is there anything that you are particularly satisfied or dissatisfied with?

Table S2. Robust linear regression testing positive and negative sentiment scores separately for predicting future depression scores (each row represents one regression result).

Study 1	***Beta estimates***	***SE***	***t-stat***	***P***
**Initial PHQ-9 score**	0.66	0.05	12.95	**<.001**
**Human positivity score**	-0.47	0.16	-2.94	**.004**
**Initial PHQ-9 score**	0.65	0.05	12.12	**<.001**
**Human negativity score**	0.49	0.17	2.95	**.004**
**Initial PHQ-9 score**	0.70	0.05	13.94	**<.001**
**ChatGPT positivity score**	-0.32	0.16	-1.98	**.049**
**Initial PHQ-9 score**	0.66	0.05	12.09	**<.001**
**ChatGPT negativity score**	0.42	0.15	2.74	**.007**
**Initial PHQ-9 score**	0.75	0.04	17.86	**<.001**
**LIWC positivity score**	-0.01	0.08	-0.14	.886
**Initial PHQ-9 score**	0.77	0.04	17.20	**<.001**
**LIWC negativity score**	-0.22	0.09	-2.61	**.010**

Study 2	***Beta estimates***	***SE***	***t-stat***	***P***
**Initial PHQ-9 score**	0.87	0.04	22.08	**<.001**
**Human positivity score**	-0.32	0.12	-2.61	**.010**
**Initial PHQ-9 score**	0.85	0.04	21.37	**<.001**
**Human negativity score**	0.40	0.12	3.32	**.001**
**Initial PHQ-9 score**	0.87	0.04	22.93	**<.001**
**ChatGPT positivity score**	-0.29	0.12	-2.37	**.018**
**Initial PHQ-9 score**	0.84	0.04	20.79	**<.001**
**ChatGPT negativity score**	0.37	0.11	3.36	**.001**
**Initial PHQ-9 score**	0.93	0.03	28.26	**<.001**
**LIWC positivity score**	0.06	0.08	0.74	.459
**Initial PHQ-9 score**	0.90	0.04	25.16	**<.001**
**LIWC negativity score**	0.16	0.09	1.83	.068

Table S3. Robust linear regression testing for an interaction between first-person singular pronouns and language sentiment to predict future depression scores (each row represents one regression result).

Study 1	***Beta estimates***	***SE***	***t-stat***	***P***
**Initial PHQ-9 score**	0.64	0.05	12.11	**<.001**
**'I' pronouns**	-0.10	0.06	-1.59	.114
**Human sentiment score**	-0.25	0.21	-1.21	.227
**'I' pronouns * Human sentiment score**	0.00	0.02	0.06	.951
**Initial PHQ-9 score**	0.66	0.05	12.84	**<.001**
**'I' pronouns**	-0.12	0.19	-0.63	.532
**Human positivity score**	-0.48	0.43	-1.12	.265
**'I' pronouns * Human positivity score**	0.01	0.04	0.13	.896
**Initial PHQ-9 score**	0.65	0.05	12.17	**<.001**
**'I' pronouns**	-0.12	0.16	-0.75	.457
**Human negativity score**	0.42	0.39	1.09	.278
**'I' pronouns * Human negativity score**	0.00	0.04	0.09	.931

Study 2	***Beta estimates***	***SE***	***t-stat***	***P***
**Initial PHQ-9 score**	0.85	0.04	21.22	**<.001**
**'I' pronouns**	-0.03	0.05	-0.47	.636
**Human sentiment score**	-0.56	0.20	-2.78	**.006**
**'I' pronouns * Human sentiment score**	0.03	0.02	1.89	.060
**Initial PHQ-9 score**	0.87	0.04	21.98	**<.001**
**'I' pronouns**	-0.36	0.17	-2.05	**.041**
**Human positivity score**	-1.10	0.40	-2.77	**.006**
**'I' pronouns * Human positivity score**	0.07	0.04	2.03	**.044**
**Initial PHQ-9 score**	0.85	0.04	21.17	**<.001**
**'I' pronouns**	0.24	0.16	1.48	.141
**Human negativity score**	1.02	0.39	2.61	**.009**
**'I' pronouns * Human negativity score**	-0.06	0.03	-1.66	.098

Table S4. Robust linear regression testing sentiment scores for predicting future depression scores after excluding responses with at least 10 words. Participants who wrote fewer than 10 words for more than five questions were excluded (each row represents one regression result).

Study 1	***Beta estimates***	***SE***	***t-stat***	***P***
**Initial PHQ-9 score**	0.68	0.05	12.48	**<.001**
**Human sentiment score**	-0.25	0.09	-2.83	**.005**
**Initial PHQ-9 score**	0.70	0.06	12.63	**<.001**
**ChatGPT sentiment score**	-0.20	0.09	-2.29	**.023**
**Initial PHQ-9 score**	0.77	0.05	16.99	**<.001**
**LIWC sentiment score**	-0.06	0.08	-0.73	.464

Study 2	***Beta estimates***	***SE***	***t-stat***	***P***
**Initial PHQ-9 score**	0.87	0.04	21.53	**<.001**
**Human sentiment score**	-0.19	0.06	-3.05	**.003**
**Initial PHQ-9 score**	0.88	0.04	21.53	**<.001**
**ChatGPT sentiment score**	-0.17	0.06	-2.74	**.006**
**Initial PHQ-9 score**	0.94	0.04	25.83	**<.001**
**LIWC sentiment score**	-0.01	0.06	-0.21	.836

Table S5. Robust linear regression testing LIWC emotion and other subcategories including anxiety, sadness, and anger for predicting future depression scores (each row represents one regression result).

A. LIWC emotion categories.

Study 1	*Beta estimates*	*SE*	*t-stat*	*P*
**Initial PHQ-9 score**	0.77	0.05	16.89	**<.001**
**LIWC emotion score**	0.10	0.07	1.37	.172
**Initial PHQ-9 score**	0.75	0.04	17.81	**<.001**
**LIWC emotion positive score**	-0.01	0.09	-0.06	.953
**Initial PHQ-9 score**	0.78	0.04	18.01	**<.001**
**LIWC emotion negative score**	-0.31	0.09	-3.56	**<.001**
**Initial PHQ-9 score**	0.77	0.04	18.07	**<.001**
**LIWC emotion anxiety score**	-0.82	0.22	-3.71	**<.001**
**Initial PHQ-9 score**	0.74	0.04	17.90	**<.001**
**LIWC emotion sadness score**	0.83	0.50	1.65	.101
**Initial PHQ-9 score**	0.75	0.04	17.88	**<.001**
**LIWC emotion anger score**	-0.87	0.43	-2.03	**.044**

Study 2	*Beta estimates*	*SE*	*t-stat*	*P*
**Initial PHQ-9 score**	0.92	0.03	26.57	**<.001**
**LIWC emotion score**	-0.01	0.08	-0.08	.937
**Initial PHQ-9 score**	0.93	0.03	28.36	**<.001**
**LIWC emotion positive score**	0.12	0.13	0.90	.368
**Initial PHQ-9 score**	0.91	0.03	26.57	**<.001**
**LIWC emotion negative score**	0.08	0.10	0.80	.424
**Initial PHQ-9 score**	0.92	0.03	28.23	**<.001**
**LIWC emotion anxiety score**	0.25	0.22	1.13	.259
**Initial PHQ-9 score**	0.91	0.03	26.51	**<.001**
**LIWC emotion sadness score**	0.41	0.38	1.09	.277
**Initial PHQ-9 score**	0.92	0.03	28.72	**<.001**
**LIWC emotion anger score**	-0.08	0.52	-0.16	.876

B. LIWC emotion categories after controlling for LIWC sentiment scores.

Study 1	*Beta estimates*	*SE*	*T-stat*	*P*
**Initial PHQ-9 score**	0.77	0.05	16.51	**<.001**
**LIWC sentiment score**	-0.09	0.13	-0.70	.487
**LIWC emotion score**	0.19	0.15	1.28	.201
**Initial PHQ-9 score**	0.76	0.05	16.68	**<.001**
**LIWC sentiment score**	0.10	0.08	1.25	.214
**LIWC emotion positive score**	-0.11	0.12	-0.86	.392

**Initial PHQ-9 score**	0.77	0.05	16.96	**<.001**
**LIWC sentiment score**	-0.03	0.07	-0.38	.708
**LIWC emotion negative score**	-0.34	0.12	-2.96	**.004**
**Initial PHQ-9 score**	0.78	0.05	17.02	**<.001**
**LIWC sentiment score**	0.04	0.06	0.58	.566
**LIWC emotion anxiety score**	-0.76	0.25	-3.06	**.003**
**Initial PHQ-9 score**	0.75	0.05	16.40	**<.001**
**LIWC sentiment score**	0.04	0.06	0.72	.471
**LIWC emotion sadness score**	0.88	0.51	1.72	.088
**Initial PHQ-9 score**	0.76	0.05	16.42	**<.001**
**LIWC sentiment score**	0.04	0.07	0.60	.548
**LIWC emotion anger score**	-0.79	0.49	-1.62	.107

Study 2	***Beta estimates***	***SE***	***t-stat***	***P***
**Initial PHQ-9 score**	0.92	0.04	25.64	**<.001**
**LIWC sentiment score**	-0.10	0.10	-1.04	.298
**LIWC emotion score**	0.11	0.13	0.81	.419
**Initial PHQ-9 score**	0.91	0.04	25.48	**<.001**
**LIWC sentiment score**	-0.09	0.07	-1.39	.166
**LIWC emotion positive score**	0.24	0.16	1.53	.128
**Initial PHQ-9 score**	0.91	0.04	25.51	**<.001**
**LIWC sentiment score**	-0.01	0.07	-0.18	.856
**LIWC emotion negative score**	0.06	0.13	0.49	.624
**Initial PHQ-9 score**	0.91	0.04	25.65	**<.001**
**LIWC sentiment score**	-0.02	0.06	-0.30	.766
**LIWC emotion anxiety score**	0.23	0.23	0.96	.337
**Initial PHQ-9 score**	0.90	0.04	24.65	**<.001**
**LIWC sentiment score**	-0.02	0.06	-0.41	.682
**LIWC emotion sadness score**	0.37	0.39	0.95	.341
**Initial PHQ-9 score**	0.91	0.04	25.68	**<.001**
**LIWC sentiment score**	-0.04	0.06	-0.69	.493
**LIWC emotion anger score**	-0.15	0.53	-0.27	.784

C. LIWC emotion categories after controlling for human sentiment scores.

Study 1	***Beta estimates***	***SE***	***t-stat***	***P***
**Initial PHQ-9 score**	0.64	0.05	11.93	**<.001**
**Human sentiment score**	-0.39	0.09	-4.26	**<.001**
**LIWC emotion score**	0.24	0.07	3.71	**<.001**
**Initial PHQ-9 score**	0.64	0.05	12.11	**<.001**
**Human sentiment score**	-0.30	0.09	-3.30	**.001**
**LIWC emotion positive score**	0.09	0.09	1.03	.307
**Initial PHQ-9 score**	0.65	0.05	12.21	**<.001**

**Human sentiment score**	-0.32	0.09	-3.63	**<.001**
**LIWC emotion negative score**	-0.34	0.09	-3.97	**<.001**
**Initial PHQ-9 score**	0.65	0.05	12.02	**<.001**
**Human sentiment score**	-0.28	0.09	-3.17	**.002**
**LIWC emotion anxiety score**	-0.81	0.21	-3.78	**<.001**
**Initial PHQ-9 score**	0.64	0.05	12.05	**<.001**
**Human sentiment score**	-0.25	0.09	-2.84	**.005**
**LIWC emotion sadness score**	0.62	0.50	1.24	.216
**Initial PHQ-9 score**	0.62	0.05	11.70	**<.001**
**Human sentiment score**	-0.31	0.09	-3.57	**<.001**
**LIWC emotion anger score**	-1.19	0.39	-3.07	**.003**

Study 2	***Beta estimates***	***SE***	***t-stat***	***P***
**Initial PHQ-9 score**	0.86	0.04	21.39	**<.001**
**Human sentiment score**	-0.23	0.07	-3.44	**.001**
**LIWC emotion score**	0.12	0.08	1.43	.154
**Initial PHQ-9 score**	0.85	0.04	21.25	**<.001**
**Human sentiment score**	-0.23	0.07	-3.59	**<.001**
**LIWC emotion positive score**	0.27	0.14	1.98	**.049**
**Initial PHQ-9 score**	0.85	0.04	21.13	**<.001**
**Human sentiment score**	-0.20	0.07	-2.99	**.003**
**LIWC emotion negative score**	-0.02	0.10	-0.21	.835
**Initial PHQ-9 score**	0.85	0.04	21.25	**<.001**
**Human sentiment score**	-0.19	0.06	-2.91	**.004**
**LIWC emotion anxiety score**	0.13	0.22	0.58	.563
**Initial PHQ-9 score**	0.85	0.04	20.81	**<.001**
**Human sentiment score**	-0.19	0.06	-2.90	**.004**
**LIWC emotion sadness score**	0.18	0.38	0.48	.633
**Initial PHQ-9 score**	0.85	0.04	21.28	**<.001**
**Human sentiment score**	-0.20	0.06	-3.08	**.002**
**LIWC emotion anger score**	-0.25	0.52	-0.48	.628

D. LIWC emotion categories calculated from a concatenated block of text responses.

Study 1	***Beta estimates***	***SE***	***t-stat***	***P***
**Initial PHQ-9**	0.76	0.05	16.79	**<.001**
**Block-level LIWC emotion score**	0.03	0.10	0.27	.790
**Initial PHQ-9 score**	0.76	0.04	17.69	**<.001**
**Block-level LIWC emotion positive score**	-0.01	0.13	-0.04	.967
**Initial PHQ-9 score**	0.76	0.04	17.67	**<.001**
**Block-level LIWC emotion negative score**	-0.08	0.15	-0.54	.593
**Initial PHQ-9 score**	0.76	0.04	18.85	**<.001**

**Block-level LIWC emotion anxiety score**	0.09	0.27	0.32	.749
**Initial PHQ-9 score**	0.74	0.04	17.84	**<.001**
**Block-level LIWC emotion sadness score**	0.66	0.55	1.21	.229
**Initial PHQ-9 score**	0.76	0.04	18.67	**<.001**
**Block-level LIWC emotion anger score**	0.01	0.28	0.05	.960

Study 2	***Beta estimates***	***SE***	***t-stat***	***P***
**Initial PHQ-9 score**	0.93	0.03	27.00	**<.001**
**Block-level LIWC emotion score**	0.04	0.08	0.44	.660
**Initial PHQ-9 score**	0.93	0.03	28.44	**<.001**
**Block-level LIWC emotion positive score**	0.10	0.14	0.74	.461
**Initial PHQ-9 score**	0.92	0.03	26.88	**<.001**
**Block-level LIWC emotion negative score**	0.00	0.11	0.01	.988
**Initial PHQ-9 score**	0.92	0.03	29.61	**<.001**
**Block-level LIWC emotion anxiety score**	0.18	0.23	0.78	.435
**Initial PHQ-9 score**	0.91	0.03	26.88	**<.001**
**Block-level LIWC emotion sadness score**	0.29	0.37	0.78	.435
**Initial PHQ-9 score**	0.92	0.03	28.65	**<.001**
**Block-level LIWC emotion anger score**	-0.12	0.46	-0.26	.798

Table S6. Spearman correlation *ρ* between ChatGPT (GPT-3.5 and GPT-4) sentiment ratings and LIWC with baseline mood parameter and emotional reactivity.

	**Baseline mood parameter**			**Emotional reactivity**		
	ChatGPT (GPT-3.5)	ChatGPT (GPT-4)	LIWC	ChatGPT (GPT-3.5)	ChatGPT (GPT-4)	LIWC
Study 1	ρ = 0.38 **(P < .001)**	ρ = 0.38 **(P < .001)**	ρ = 0.32 **(P < .001)**	ρ = -0.05 (P = .530)	ρ = -0.06 (P = .460)	ρ = -0.05 (P = .487)
Study 2	ρ = 0.29 **(P < .001)**	ρ = 0.28 **(P < .001)**	ρ = 0.27 **(P < .001)**	ρ = -0.04 (P = .464)	ρ = -0.01 (P = .801)	ρ = -0.01 (P = .883)

Table S7. Sentiment ratings by human raters, ChatGPT (GPT-3.5), and LIWC for an example participant from Study 2 who had a depression (PHQ-9) score of 17 at baseline and a PHQ-9 score of 20 at follow-up. Each human sentiment rating is an average score from two independent human raters.

**Question number**	**Example response**	**Human raters**		**ChatGPT 3.5**		**LIWC**	
		Pos	Neg	Pos	Neg	Pos	Neg
**1**	I have been feeling a sense of disparity, anger, laziness, complacency leading to motions of failure and dissapointment.	1	9.5	2	8	0	11.11
**2**	I have found little interest or motivation to do anything that is meaninful to me	0.5	9	2	8	0	0
**3**	My diet has been very varied I consume a lot in the meals i eat mostly but am eating at random times and not many times like usual	4.5	5	4	5	0	0
**4**	Horrible, waking up early going to bed late. less then 5 hours every night occasional naps and just out of timing circadian rythom	0	10	2	9	0	4.35
**5**	Easier which is to say that i have been finding it hard to be and stay active	2.5	6.5	2	8	5.88	0
**6**	Decreased from a usual standpoint, spikes of energy at times but generally low	4	7.5	3	6	0	0
**7**	Feeling lazy and lethargic in my tendancies, very complacent in my life also	4.5	6	2	8	0	15.38
**8**	My concentration has probably been at an all time low decision making as well at a recent low point, making ill informed unthought decsisions	0	8.5	2	8	4.17	0
**9**	My attitudes to important things my work ethic and consistency as well as commitment	8.5	1	7	0	14.29	0
**Total mean score**		2.83	7.00	2.89	6.67	2.70	3.43
**Total mean composite score**		-4.17		-3.78		-0.72	

Table S8. Inter-rater Pearson correlation coefficients *R* between the first and second human sentiment ratings per prompt in Studies 1 and 2. All correlation coefficients were significant at *P* < .005.

Question number	Positive sentiment score		Negative sentiment score	
	Study 1	Study 2	Study 1	Study 2
**1**	0.67	0.66	0.70	0.60
**2**	0.58	0.61	0.58	0.54
**3**	0.31	0.36	0.53	0.43
**4**	0.41	0.59	0.43	0.54
**5**	0.24	0.26	0.35	0.39
**6**	0.65	0.57	0.56	0.55
**7**	0.78	0.70	0.78	0.76
**8**	0.39	0.55	0.42	0.54
**9**	0.73	0.69	0.71	0.65

Reference

1. Nook, E. C., Hull, T. D., Nock, M. K. & Somerville, L. H. Linguistic measures of psychological distance track symptom levels and treatment outcomes in a large set of psychotherapy transcripts. *Proc. Natl. Acad. Sci.* **119**, e2114737119 (2022).
